# Design, synthesis, drug-likeness, and molecular dynamics evaluations of monobrominated amathamide G analogs as potential ligands for D2-like dopamine receptors

**DOI:** 10.1007/s40203-026-00741-1

**Published:** 2026-09-06

**Authors:** Angélica Navarrete-Gutiérrez, Octavio Frías-Aviña, Julio Montes-Ávila, Raúl Argüello-García, Edar O. Pech-Santiago, Gerardo Aguirre-Hernández

**Affiliations:** 1 Centro de Graduados e Investigación en Química, Tecnológico Nacional de México/IT de Tijuana, Tijuana, B.C. Mexico; 2 Facultad de Ciencias Químico Biológicas, Universidad Autónoma de Sinaloa, Culiacán, Sin. Mexico; 3 Departamento de Genética y Biología Molecular, Centro de Investigación y de Estudios Avanzados del Instituto Politécnico Nacional, Mexico City, Mexico

**Keywords:** D2-like dopamine receptors, Amathamide G derivatives, Brominated alkaloids, Pharmacological predictions, Molecular docking, Molecular dynamics simulations

## Abstract

Dopamine receptors (DRs) are key modulators of physiological and behavioral responses in the central nervous system (CNS), playing a major role in psychotic disorders. Among these, the D2-like members D2R, D3R and D4R are particularly attractive therapeutic targets for the development of novel compounds derived from both synthetic and natural sources. The marine bryozoan *Amathia* produces alkaloids known as amathamides (A-H), among which the tribrominated amathamide G (AM-G) stands out as a promising scaffold. Its benzene–aliphatic linker–pentacyclic structure is reminiscent of standard dopamine antagonists such as eticlopride (ETI). In this study, a debrominated (AM-0) and monobrominated derivatives (M4B, M5B and M6B) were successfully synthesized and purified. As an initial preclinical assessment, the drug likeness profiles of these compounds were predicted and analyzed based on physicochemical properties, medicinal chemistry, and pharmacokinetic parameters. The analysis revealed that debromination of AM-G improves both lipid and aqueous solubility, enhances overall drug desirability across four major medicinal chemistry rule sets, and predicts better CNS penetration, bioavailability, half-life, and clearance, while maintaining a relatively low risk of carcinogenicity. Molecular docking and molecular dynamics (MD) simulations suggested a differential affinity amongst amathamide G derivatives towards D2-like receptors. Specifically, AM-G behaved as a high-affinity putative ligand for D3R/D4R, whereas M5B and M6B showed selectivity for D2R. These findings support continued in vitro and in vivo evaluation of these compounds, particularly in models involving expression, structure-based variations, and pathologies associated with D2R-like receptors.

**Supplementary Information:**

The online version contains supplementary material available at https://doi.org/10.1007/s40203-026-00741-1.

## Introduction

The dopamine receptor (DR) family belongs to the G protein-coupled receptor (GPCR) superfamily, which is highly expressed in the central nervous system (CNS) of vertebrates. To date, five dopamine receptor subtypes have been identified and are classified into two major families: D1-like (D1R and D5R) and D2-like (D2R, D3R, and D4R) (Beaulieu and Gainetdinov 2011; Perreault et al. 2014). These receptors play a fundamental role in dopaminergic neurotransmission and differ in both their intracellular signaling pathways and physiological effects. D1-like receptors are coupled to stimulatory G-proteins (G_s_), which activate adenylate cyclase and increase intracellular cAMP levels. In contrast, D2-like receptors are coupled with inhibitory G-proteins (G_i_ and G_0_), thereby suppressing adenylate cyclase activity and consequently reducing cAMP levels (Beaulieu and Gainetdinov 2011). Dopamine receptors are involved in a wide range of neurophysiological functions, including motor control (Ryczko and Dubuc 2023), mood regulation, cognitive modulation (Ott and Nieder 2019), and memory processing (Carr et al. 2017). Consequently, they serve as key targets in the treatment of various neurodegenerative and neuropsychiatric disorders such as schizophrenia (Martel and Gatti McArthur 2020), Parkinson’s disease (Mishra et al. 2018), Alzheimer’s disease (Pan et al. 2019), substance addiction (Wise and Robble 2020), and mood disorders (Radwan et al. 2019). Notably, the roles of D2-like receptors are interrelated: all three subtypes contribute to attention and sleep regulation, with D2R also involved in locomotion, memory, and learning; D3R and D4R in cognition and impulse control, and D4R is specifically implicated in memory and fear responses (Mishra et al. 2018). Given their central role in CNS function, D2-like receptors are considered primary targets in the development of neuromodulatory drugs, particularly antipsychotic agents. These often include alkaloids—organic compounds containing at least one nitrogen atom—many of which are derived from animal or plant sources and possess prophylactic and therapeutic applications in proactive medicine.

Over recent decades, natural product chemistry has increasingly focused on secondary metabolites from marine organisms, leading to the discovery of numerous compounds with significant pharmacological potential (Molinski et al. 2009; Haque et al. 2022). Marine species are exposed to extreme environmental conditions—such as high pressure, variable temperatures, low oxygen levels, and, in deep-sea environments, total darkness—which drives the production of unique bioactive metabolites (Danovaro et al. 2014). Among these organisms, bryozoans (also known as moss animals) have been recognized as valuable sources of biologically active substances (Figuerola and Avila 2019; Ciavatta et al. 2020). Most compounds isolated from bryozoans are alkaloids, which exhibit a wide range of biological activities, including antibacterial (Walls et al. 1993), antiprotozoal (Narkowicz et al. 2004), nematocidal (Narkowicz et al. 2002), antifeedant (Montanari et al. 1996; Sherwood et al. 1998), and reversal of drug resistance in leukemia-derived cell lines (Hashima et al. 2000). A noteworthy group of bryozoan-derived alkaloids is the amathamides, a family of brominated compounds (A–H) isolated from *Amathia* species (Blackman and Green 1987). Several members of this family exhibit significant biological activities: for instance, amathamide A possesses antibacterial properties (Ramírez-Osuna et al. 2005), while amathamides C and H display antimalarial and antitrypanosomal activities (Carroll et al. 2011). More recently, amathamide E has been predicted to interact with multiple molecular targets in triple-negative breast cancer cells (Manivannan et al. 2024). Despite these promising findings, a substantial gap of knowledge remains regarding the potential CNS-modulatory effects of amathamides and other related marine natural products.

The chemical structure of amathamides is characterized by the presence of an N-methyl-L-proline ring and a halogenated, methoxylated aromatic ring, connected through an enamide group. Their classification is determined by the substitution pattern on the aromatic ring and the nitrogen atom of the enamide moiety. Reports on the chemical synthesis of amathamides remain limited; to date, only amathamides A, B, D, F, and related analogs have been prepared (Ramírez Osuna et al. 2002; Ramírez-Osuna et al. 2005; Ramírez-Osuna et al. 2011; Khan and Ahmad 2012; Ahmad et al. 2015). Amathamides A and B were synthesized previously by our research group through removal of a double-bond protecting group to generate the enamide functionality. Amathamides D and F were synthesized by different ways: Amathamide D via coupling with N-methyl-L-proline (Ahmad et al. 2015), while amathamide F through a Pd-catalyzed coupling between a β-substituted vinyl acetate and N-methyl-L-prolinamide (Khan and Ahmad 2012). Biological studies have further demonstrated that the antimicrobial activity of amathamide A and its analogs depend on the presence of bromine substituents (Ramírez-Osuna et al. 2005).

Building on prior work involving the synthesis and biological evaluation of amathamides, we hypothesize that bromine substitution plays a key role in modulating their bioactivity. The objective of this study was to synthesize monobrominated derivatives of the tribrominated amathamide G (AM-G) and to assess their potential interactions with dopamine D2R, D3R, and D4R receptors (D2-like family) using both analytical and predictive bioinformatics approaches. These findings may provide a framework for future in vitro and in vivo studies, supporting structure-based drug discovery strategies for the development of improved pharmacological treatments for neurodegenerative and psychiatric disorders.

## Experimental

### Materials and instruments

All reagents were purchased commercially and used without further purification. ^1^H and ^13^C NMR spectra were recorded on a Bruker Avance III HD 400 MHz spectrometer using CDCl_3_ as the solvent. Chemical shifts (δ) are reported in parts per million (ppm) and coupling constants (*J*) are given in hertz (Hz). Products were purified by column chromatography on silica gel (60 Å, 200−400 mesh). Reaction progress was monitored by thin–layer chromatography (TLC), with spots visualized under UV light (254 nm). Infrared spectra were recorded on a Spectrum 400 spectrophotometer. Mass spectra were obtained on an Agilent Technologies 5975C instrument by direct insertion. Melting points were determined using a Fisher–Johns melting point apparatus.

### Synthesis and characterization details

#### Design of structural monobrominated derivatives of amathamide G

**Fig. 1:**
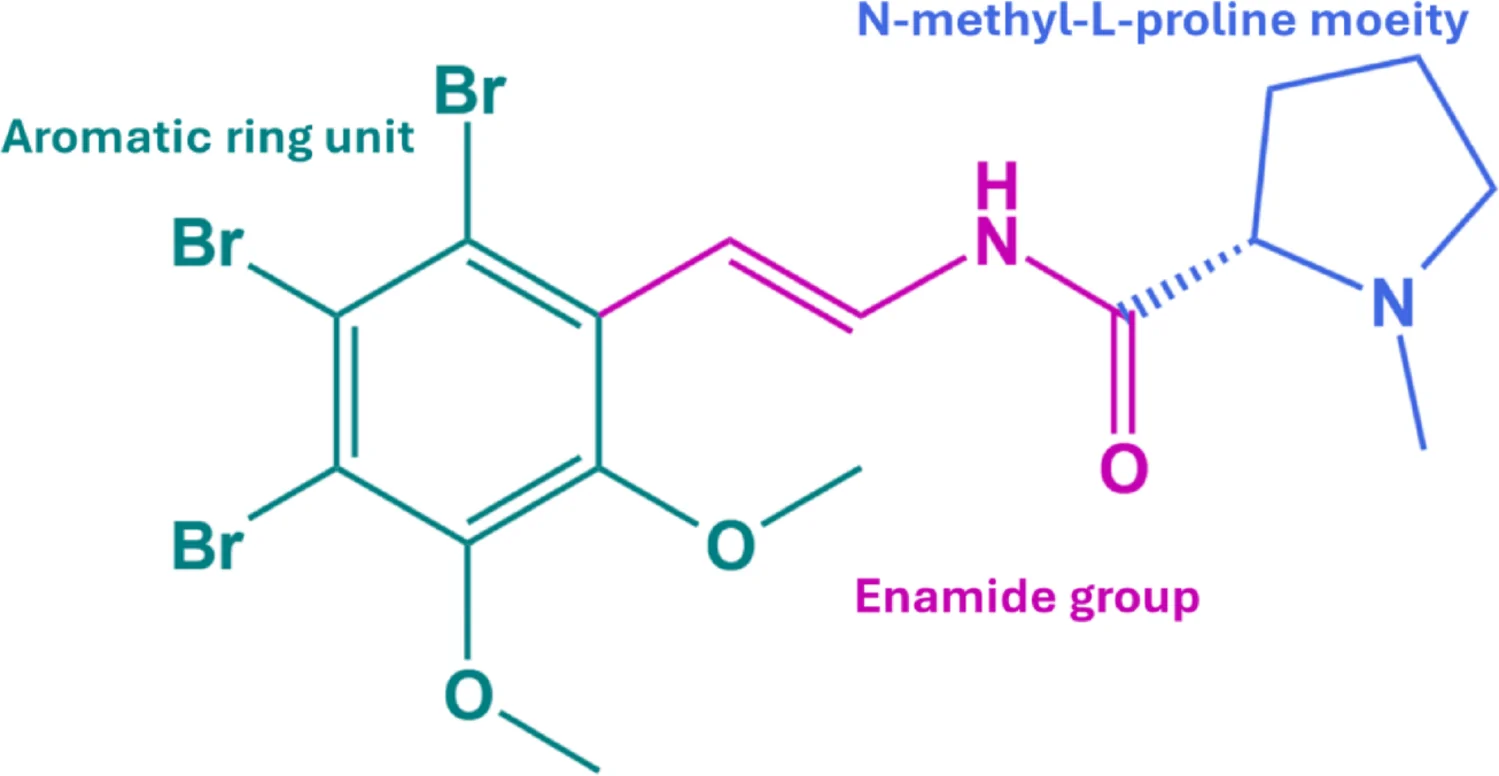
Common moieties of the monobrominated analogues of amathamide G

The structure of amathamide G consists of a tribrominated aromatic ring, an N-methyl-L-proline moiety, and an enamide group. To obtain monobrominated derivatives of amathamide G, these three structural features must be addressed (Fig. 1). The aromatic ring unit is derived from 2,3-dimethoxybenzaldehyde and its monobrominated analogues, which serve as starting materials for the synthetic route. The Henry reaction allows for the synthesis of a β-nitrostyrene from benzaldehyde, which can then undergo a Michael addition with thiophenol. The nitro group is particularly useful, as it can be reduced to an amine; this amine is then coupled with N-methyl-L-proline to install that specific moiety. Finally, while few methodologies have been reported for synthesizing the enamide group in amathamides and their derivatives, the elimination of a sulfoxide was a convenient approach for this work.

#### Procedure for synthesis of compounds 2a-2d

To a solution of aldehyde **1a** (2.00 g, 12.0 mmol) in glacial acetic acid (30 mL), ammonium acetate (2.31 g, 30.0 mmol) and nitromethane (2.0 mL) was added. The reaction mixture was heated under reflux for 3 h and then cooled to room temperature. The solvent was removed under reduced pressure, and distilled water (50 mL) was added. The product was extracted with dichloromethane (3 × 30 mL), and the combined organic layers were washed with brine and water, dried over anhydrous sodium sulfate, and concentrated under reduced pressure to afford nitroolefin **2a**, obtained in sufficient purity for use in the subsequent step.

***(E)− 1,2-dimethoxy-3-(2-nitrovinyl)benzene (2a)***. Yellow solid (1.91 g, 76%); m. p. 86–88 °C; FTIR (ATR): 3109, 3008, 2832, 1628, 1570, 1510 cm^−1^; ^1^H NMR (400 MHz, CDCl_3_): δ 8.16 (d, *J* = 13.8 Hz, 1H), 7.72 (d, *J* = 13.7 Hz, 1H), 7.17–6.88 (m, 3H), 3.90 (s, 3H), 3.88 (s, 3H). ^13^C NMR (100 MHz, CDCl_3_): δ 153.25, 149.55, 138.56, 134.70, 124.53, 124.18, 121.45, 116.04, 61.17, 56.02; EI-MS (m/z): M^+^ 209.1.

***(E)− 1-bromo-3,4-dimethoxy-2-(2-nitrovinyl)benzene (2b)***. Yellow solid (1.88 g, 80%); m. p. 118 °C; FTIR (ATR): 3155, 2941, 1620, 1522, 1462 cm^−1^; ^1^H NMR (400 MHz, CDCl_3_): δ 8.27 (d, *J* = 13.6 Hz, 1H), 7.98 (d, *J* = 13.6 Hz, 1H), 7.35 (d, *J* = 8.9 Hz, 1H), 6.88 (d, *J* = 8.8 Hz, 1H), 3.88 (s, 3H), 3.87 (s, 3H); ^13^C NMR (100 MHz, CDCl_3_): δ 152.51, 150.38, 141.60, 134.07, 128.76, 124.16, 117.13, 116.21, 60.53, 56.26; EI-MS (m/z): M^+^ 287.0.

***(E)− 5-bromo-1,2-dimethoxy-3-(2-nitrovinyl)benzene (2c)***. Yellow solid (1.67 g, 71%); m. p. 126–127 °C; FTIR (ATR): 3017, 2948,1719, 1625, 1570, 1498 cm^−1^; ^1^H NMR (400 MHz, CDCl_3_): δ 8.07 (d, *J* = 13.8 Hz, 1H), 7.67 (d, *J* = 13.8 Hz, 1H), 7.17 (d, *J* = 2.2 Hz, 1H), 7.11 (d, *J* = 2.2 Hz, 1H), 3.89 (s, 3H), 3.88 (s, 3H); ^13^C NMR (100 MHz, CDCl_3_): δ 153.88, 148.69, 139.30, 133.19, 125.53, 123.54, 118.97, 116.81, 61.35, 56.36; EI-MS (m/z): M^+^ 287.0.

***(E)− 1-bromo-2,3-dimethoxy-4-(2-nitrovinyl)benzene (2d)***. Yellow solid (1.72 g, 73%); m. p. 88–89 °C; FTIR (ATR): 3102, 2982, 2939, 1630, 1560 cm^−1^; ^1^H NMR (400 MHz, CDCl_3_): δ 8.08 (d, *J* = 13.7 Hz, 1H), 7.74 (d, *J* = 13.8 Hz, 1H), 7.33 (d, *J* = 8.5 Hz, 1H), 7.10 (d, *J* = 8.6 Hz, 1H), 3.97 (s, 3H), 3.88 (s, 3H); ^13^C NMR (100 MHz, CDCl_3_): δ 154.18, 151.24, 138.81, 133.78, 128.61, 125.79, 124.36, 122.67, 61.37, 60.78; EI-MS (m/z): M^+^ 287.0.

#### Procedure for synthesis of compounds 3a-3d

A solution of nitroolefin **2a** (1.00 g, 4.78 mmol) in thiophenol (2.0 mL) was stirred at room temperature for 3 h. Excess thiophenol was removed by filtration through a silica plug using petroleum ether. The product was dissolved in dichloromethane, and the solvent was evaporated under reduced pressure to afford compound **3a**, obtained in sufficient purity for use in the subsequent step.

***(1-(2,3-dimethoxyphenyl)− 2-nitroethyl)(phenyl)sulfane (3a)***. Orange liquid (1.45 g, 95%); FTIR (ATR): 30,256, 2938, 2832, 1548, 1479, 1370, 1267, 1221, 1084 cm^−1^; ^1^H NMR (400 MHz, CDCl_3_): δ 7.49–7.39 (m, 2H), 7.38–7.25 (m, 3H), 6.99 (t, *J* = 8.0 Hz, 1H), 6.88 (dd, *J* = 8.2, 1.5 Hz, 1H), 6.74 (dd, *J* = 7.8, 1.5 Hz, 1H), 5.28 (dd, *J* = 9.1, 6.7 Hz, 1H), 4.91 (dd, *J* = 13.1, 9.1 Hz, 1H), 4.76 (dd, *J* = 13.1, 6.7 Hz, 1H), 3.93 (s, 3H), 3.86 (s, 3H); ^13^C NMR (100 MHz, CDCl_3_): δ 153.02, 147.13, 133.58, 133.01, 130.28, 129.39, 128.65, 124.17, 119.51, 112.92, 78.16, 61.19, 55.91, 44.82; EI-MS (m/z): M^+^ 319.2.

***(1-(6-bromo-2,3-dimethoxyphenyl)− 2 nitroethyl)(phenyl)sulfane (3b)***. Colorless solid (1.36 g, 98%); m. p. 74–75 °C; FTIR (ATR): 3056, 2938, 2835, 1558, 1464, 1257, 1082, 1002 cm^−1^; ^1^H NMR (400 MHz, CDCl_3_): δ 7.58–7.53 (m, 2H), 7.36–7.31 (m, 3H) 7.26 (d, *J* = 8.9 Hz, 1H), 6.77 (d, *J* = 8.9 Hz, 1H), 5.46 (dd, *J* = 8.3, 7.0 Hz, 1H), 5.15 (dd, *J* = 13.2, 8.3 Hz, 1H), 4.90 (dd, *J* = 13.2, 7.0 Hz, 1H), 4.04 (s, 3H), 3.86 (s, 3H); ^13^C NMR (100 MHz, CDCl_3_): δ 152.48, 149.25, 134.07, 133.45, 130.70, 129.43, 128.63, 127.88, 115.02, 114.03, 77.82, 61.89, 56.10, 50.10; EI-MS (m/z): M^+2^ 399.0.

***(1-(5-bromo-2,3-dimethoxyphenyl)− 2-nitroethyl)(phenyl)sulfane (3c)***. Orange liquid (1.34 g, 97%); FTIR (ATR): 2977, 2892, 1474, 1373, 1226, 993 cm^−1^; ^1^H NMR (400 MHz, CDCl_3_): δ 7.45–7.40 (m, 2H), 7.35–7.31 (m, 3H), 6.98 (d, *J* = 2.2 Hz, 1H), 6.83 (d, *J* = 2.2 Hz, 1H), 5.20 (dd, *J* = 9.1, 6.7 Hz, 1H), 4.84 (dd, *J* = 13.2, 9.0 Hz, 1H), 4.73 (dd, *J* = 13.3, 6.7 Hz, 1H), 3.89 (s, 3H), 3.85 (s, 3H); ^13^C NMR (100 MHz, CDCl_3_): δ 153.64, 146.33, 133.89, 132.34, 131.98, 129.49, 129.00, 122.40, 116.41, 116.27, 77.84, 61.27, 56.20, 44.44; EI-MS (m/z): M^+2^ 399.1.

***(1-(4-bromo-2,3-dimethoxyphenyl)− 2-nitroethyl)(phenyl)sulfane (3d)***. Brown liquid (1.15 g, 83%); FTIR (ATR): 2849, 1548, 1457, 1402, 1373, 1269, 1000 cm^−1^; ^1^H NMR (400 MHz, CDCl_3_): δ 7.44–7.38 (m, 2H), 7.34–7.29 (m, 3H) 7.21 (d, *J* = 8.4 Hz, 1H), 6.74 (d, *J* = 8.4 Hz, 1H), 5.20 (dd, *J* = 9.3, 6.6 Hz, 1H), 4.87 (dd, *J*= 9.33, 13.15 Hz, 1H), 4.75 (dd, 6.56, 13.16 Hz, 1H), 3.96 (s, 3H), 3.86 (s, 3H); ^13^C NMR (100 MHz, CDCl_3_): δ 152.20, 150.73, 133.86, 132.20, 130.42, 129.43, 128.91, 127.86, 123.63, 118.42, 77.70, 61.29, 60.49, 44.44. EI-MS (m/z): M^+^ 399.1.

#### Procedure for synthesis of compounds 4a-4d

To a suspension of compound **3a** (0.50 g, 1.56 mmol) and Zn powder (1.02 g, 15.6 mmol) in glacial acetic acid (10 mL), conc. hydrochloric acid (3.5 mL) was added dropwise. The mixture was stirred at room temperature for 1 h and then filtered through a pad of silica gel. Water (30 mL) was added to the filtrate, neutralized with 2 N ammonium hydroxide, and extracted with dichloromethane (3 × 30 mL). The combined organic layers were dried over anhydrous sodium sulfate and concentrated under reduced pressure to give amine **4a**, which was used in the next step without further purification.

#### Procedure for synthesis of compounds 5a-5d

To a solution of *N*-methyl-L-proline (0.25 g, 1.94 mmol) in anhydrous dichloromethane (10 mL), 1-hydroxybenzotriazole (0.35 g, 2.60 mmol), EDC hydrochloride (0.45 g, 2.35 mmol), N,N-diisopropylethylamine (0.5 mL) and 4 Å molecular sieves was added. The mixture was stirred at 0 °C for 30 min and then allowed to warm at room temperature. A solution of amine **4a** (0.45 g, 1.56 mmol) in anhydrous dichloromethane (0.5 mL) was added, and the reaction mixture was stirred at room temperature for 18 h. The reaction was quenched with 1 N hydrochloric acid, and distilled water was added. The organic layer was separated, washed with saturated aqueous sodium bicarbonate solution and brine, dried over anhydrous sodium sulfate and concentrated under reduced pressure. The residue was purified by silica gel column chromatography (eluent: acetonitrile) to afford enamine **5a**.

***(2S)-N-(2-(2,3-dimethoxyphenyl)− 2-(phenylthio)ethyl)− 1-methylpyrrolidine−2-carboxamide (5a)***. Yellow liquid (0.13 g, 21%); FTIR (ATR): 3324, 2933, 2847, 2782, 1656, 1580, 1510, 1471 cm^−1^; ^1^H NMR (CDCl_3_, 400 MHz): δ 7.47–7.37 (m, 1H), 7.36–7.28 (m, 2H), 7.21–7.09 (m, 3H), 6.94–6.88 (m, 1H), 6.82–6.72 (m, 2H), 4.81–4.71 (m, 1H), 3.77–3.73 (m, 6H), 3.69–3.59 (m, 2H), 2.92–2.85 (m, 1H), 2.75–2.66 (m, 1H), 2.22–2.12 (m, 1H), 2.12–2.06 (m, 3H), 2.05–1.92 (m, 1H), 1.71–1.40 (m, 3H); ^13^C NMR (100 MHz, CDCl_3_): δ 174.66, 174.57, 152.80, 152.79, 147.24, 147.20, 134.91, 134.86, 133.41, 133.38, 132.03, 131.97, 129.00, 128.99, 127.27, 127.23, 124.12, 124.08, 120.32, 111.87, 68.90, 61.10, 56.69, 56.67, 55.89, 46.01, 45.97, 43.19, 43.10, 41.56, 41.49, 30.97, 30.95, 24.29, 24.27; IE-MS (m/z): M^+^ 400.2.

***(2S)-N-(2-(6-bromo-2,3-dimethoxyphenyl)− 2-(phenylthio)ethyl)− 1-methylpyrrolidine−2-carboxamide (5b)***. Brown liquid (0.17 g, 28%). FTIR (ATR): 3335, 3056, 2835, 2787, 1656, 1507, 1464, 1269, 1219, 1079, 998 cm^−1^; ^1^H NMR (CDCl_3_, 400 MHz): δ 7.50–7.37 (m, 3H), 7.25–7.17 (m, 2H), 7.17–7.08 (m, 2H), 6.68–6.60 (m, 1H), 5.22–4.88 (m, 1H), 3.99–3.94 (m, 3H), 3.91–3.70 (m, 5H), 2.94–2.78 (m, 1H), 2.77–2.64 (m, 1H), 2.21–1.96 (m, 5H), 1.74–1.37 (m, 3H); ^13^C NMR (100 MHz, CDCl_3_): δ 174.65, 152.48, 149.27, 149.24, 136.80, 136.56, 133.47, 133.42, 131.07, 131.03, 130.71, 130.61, 129.04, 127.42, 127.34, 126.87, 115.69, 115.65, 113.28, 113.24, 68.80, 68.75, 61.74, 56.72, 56.63, 56.02, 51.65, 51.43, 42.94, 41.64, 41.44, 30.92, 30.84, 24.35, 24.30; IE-MS (m/z): M^+2^ 480.1.

***(2S)-N-(2-(5-bromo-2,3-dimethoxyphenyl)− 2-(phenylthio)ethyl)− 1-methylpyrrolidine−2-carboxamide (5c)***. Orange liquid (0.28 g, 45%). FTIR (ATR): 3330, 3054, 2933, 2835, 2787, 1659, 1558, 1510, 1469, 1272, 1219, 1161, 1000 cm^−1^; ^1^H NMR (CDCl_3_, 400 MHz): δ 7.61–7.50 (m, 1H), 7.42–7.35 (m, 2H), 7.30–7.18 (m, 3H), 7.03–7.00 (m, 1H), 6.93 (d, *J* = 2.04, 1H), 4.85–4.74 (m, 1H), 3.84–3.78 (m, 6H), 3.75–3.63 (m, 2H), 3.05–2.97 (m, 1H), 2.86–2.78 (m, 1H), 2.33–2.19 (m, 4H), 2.18–2.07 (m, 1H), 1.79–1.55 (m, 3H); ^13^C NMR (100 MHz, CDCl_3_): δ 174.77, 174.67, 153.40, 153.38, 146.35, 146.30, 135.22, 135.19, 134.29, 134.26, 132.18, 132.05, 129.05, 127.52, 127.46, 123.19, 116.37, 116.34, 115.19, 68.86, 61.12, 56.69, 56.68, 56.10, 45.98, 45.94, 43.05, 42.99, 41.69, 41.53, 30.98, 24.34, 24.32; IE-MS (m/z): M^+^ 477.1.

***(2S)-N-(2-(4-bromo-2,3-dimethoxyphenyl)− 2-(phenylthio)ethyl)− 1-methylpyrrolidine−2-carboxamide (5d)***. Green liquid (0.21 g, 34%). FTIR (ATR): 3330, 2936, 2844, 2872, 1656, 1507, 1454, 1269, 851 cm^−1^; ^1^H NMR (400 MHz, CDCl_3_): δ 7.50–7.39 (m, 1H), 7.31–7.24 (m, 2H), 7.21–7.11 (m, 4H), 6.81 (d, *J* = 8.5 Hz, 1H), 4.74–4.34 (m, 1H), 3.77–3.73 (m, 6H), 3.66–3.59 (m, 2H), 2.95–2.87 (m, 1H), 2.75–2.69 (m, 1H), 2.24–2.16 (m, 1H), 2.16–2.10 (m, 3H), 2.09–2.00 (m, 1H), 1.7–1.43 (m, 3H); ^13^C NMR (100 MHz, CDCl_3_): δ 174.67, 174.57, 152.34, 152.30, 150.47, 150.46, 134.06, 133.99, 133.69, 133.64, 132.66, 132.62, 129.06, 129.05, 127.84, 127.81, 127.69, 127.66, 124.42, 117.03, 117.01, 68.94, 68.93, 61.24, 61.23, 60.46, 56.70, 56.69, 45.97, 45.92, 42.80, 42.71, 41.70, 41.59, 31.00, 24.34, 24.31; IE-MS (m/z): M^+2^ 480.1.

#### Procedure for synthesis of compounds 6a-6d

A solution of enamine **5a** (0.12 g, 0.30 mmol) in acetic acid/acetonitrile (1:1, 2.0 mL) was treated with a solution of sodium periodate (0.13 g, 0.60 mmol) in acetic acid/water (1:1, 2.0 mL), and the mixture was stirred at room temperature for 24 h. The reaction mixture was diluted with dichloromethane (10 mL), filtered, neutralized with 2 N ammonium hydroxide, and extracted with dichloromethane (3 × 15 mL). The combined organic layers were dried over anhydrous sodium sulfate and concentrated under reduced pressure (without heating) to give the corresponding sulfoxide, which was used in the next step without further purification. The sulfoxide was dissolved in xylene (2.0 mL), and potassium carbonate (0.08 mg, 0.60 mmol) was added. The mixture was heated under reflux for 1 h and then cooled to room temperature. The solvent was removed under reduced pressure, and the residue was purified by silica gel column chromatography (eluent: acetonitrile) to afford the amathamide analog **6a**.

***(S,E)-N-(2,3-dimethoxystyryl)− 1-methylpyrrolidine−2-carboxamide (6a)***. Yellow liquid (0.044 g, 51%); $[\alpha ]_{D}^{{25}}$ − 53.41(c0.920,CHCl_3_); FTIR (ATR): 3277, 3068, 2972, 2931, 1642, 1474, 1260, 1065, 995 cm^−1^; ^1^H NMR (CDCl_3_, 400 MHz): δ 9.16 (d, *J* = 11.6 Hz, 1H), 7.50 (dd, *J* = 14.8, 11.4 Hz, 1H), 7.08 (dd, *J* = 7.9, 1.4 Hz, 1H), 6.98 (t, *J* = 8.0 Hz, 1H), 6.74 (dd, *J* = 8.1, 1.5 Hz, 1H), 6.46 (d, *J* = 14.8 Hz, 1H), 3.85 (s, 3H), 3.81 (s, 3H), 3.19–3.13 (m, 1H), 2.99 (dd, J= 10.31, 5.22 Hz, 1H), 2.41 (s, 3H), 2.39–2.36 (m, 1H), 2.29–2.21 (m, 1H), 1.93–1.85 (m, 1H), 1.83–1.74 (m, 2H); ^13^C NMR (100 MHz, CDCl_3_): δ 172.43, 153.07, 146.06, 130.47, 124.27, 123.36, 117.40, 110.51, 107.54, 68.86, 60.89, 56.82, 55.84, 42.05, 31.19, 24.59; IE-MS (m/z): M^+^ 290.1.

***(S,E)-N-(6-bromo-2,3-dimethoxystyryl)− 1-methylpyrrolidine−2-carboxamide (6b)***. Colorless solid (0.038 g, 44%); m. p. 131–132 °C. $[\alpha ]_{D}^{{25}}$ − 102.7(c0.680,CHCl_3_); FTIR (ATR): 3217, 2953, 2926, 2830, 2772, 1673, 1628, 1493, 1454, 1411, 1346, 1253, 1089, 1007 cm^−1^; ^1^H NMR (CDCl_3_, 400 MHz): δ 9.06 (d, *J* = 11.6 Hz, 1H), 7.83 (dd, *J* = 14.6, 11.6 Hz, 1H), 7.18 (d, *J* = 8.9 Hz, 1H), 6.56 (d, *J* = 8.8 Hz, 1H), 6.23 (d, *J* = 14.6 Hz, 1H), 3.77 (s, 3H), 3.75 (s, 3H), 3.14–3.08 (m, 1H), 2.93 (dd, *J* = 10.2, 5.3 Hz, 1H), 2.38–2.29 (m, 4H), 2.23–2.13 (m, 1H), 1.90–1.81 (m, 1H), 1.78–1.70 (m, 2H); ^13^C NMR (100 MHz, CDCl_3_): δ 172.32, 152.76, 147.88, 129.88, 128.35, 128.21, 114.73, 111.56, 108.30, 69.01, 59.95, 56.85, 56.19, 42.12, 31.14, 24.56; IE-MS (m/z): M^+^ 368.2.

***(S,E)-N-(5-bromo-2,3-dimethoxystyryl)− 1-methylpyrrolidine−2-carboxamide (6c)***. Orange liquid (0.051 g, 46%). $[\alpha ]_{D}^{{25}}$ − 74.83(c1.013,CHCl_3_); FTIR (ATR): 3301, 3073, 2936, 2779, 1673, 1644, 1491, 1279, 1228, 983 cm^−1^; ^1^H NMR (CDCl_3_, 400 MHz): δ 9.22 (d, *J* = 10.36 Hz, 1H), 7.46 (dd, *J* = 14.73, 11.47 Hz, 1H), 7.20 (d, *J* = 2.24, 1H), 6.83 (d, *J* = 2.25 Hz, 1H), 6.36 (d, *J* = 14.76 Hz), 3.82 (s, 3H), 3.77 (s, 3H), 3.21–3.13 (m, 1H), 3.08–2.99 (m, 1H), 2.47–2.38 (m, 4H), 2.29–2.20 (m, 1H), 1.95–1.86 (m, 1H), 1.84–1.75 (m, 2H); ^13^C NMR (100 MHz, CDCl_3_): δ 172.29, 153.68, 145.10, 132.07, 124.32, 120.11, 116.91, 113.75, 106.33, 68.78, 60.89, 56.78, 56.10, 41.95, 31.13, 24.55; IE-MS (m/z): M^+^ 368.1.

***(S,E)-N-(4-bromo-2,3-dimethoxystyryl)− 1-methylpyrrolidine−2-carboxamide (6d)***. Yellow liquid (0.064 g, 58%); $[\alpha ]_{D}^{{25}}$ − 56.50(c1.020,CHCl_3_); FTIR (ATR): 3227, 3063, 2943, 2849, 1637, 1454, 1231, 998, 844 cm^−1^; ^1^H NMR (400 MHz, CDCl_3_): δ 9.16 (d, *J* = 11.4 Hz, 1H), 7.50 (dd, *J* = 14.8, 11.4 Hz, 1H), 7.18 (d, *J* = 8.6 Hz, 1H), 7.06 (d, *J* = 8.6 Hz, 1H), 6.32 (d, *J* = 14.8 Hz, 1H), 3.85 (s, 3H), 3.84 (s, 3H), 3.18–3.11 (m, 1H), 2.99 (dd, *J* = 10.3, 5.2 Hz, 1H), 2.43–2.35 (m, 4H), 2.30–2.20 (m, 1H), 1.93–1.83 (m, 1H), 1.83–1.72 (m, 2H); ^13^C NMR (100 MHz, CDCl_3_): δ 172.42, 150.97, 150.83, 130.69, 128.03, 123.89, 121.46, 115.29, 106.83, 68.82, 60.98, 60.66, 56.79, 42.01, 31.16, 24.56; IE-MS (m/z): M^+^ 368.2.

### Computational studies

#### Molecular docking

The synthesized compounds were sketched in MolView Web application (Smith 1995) and further geometry-optimized using Avogadro software (Hanwell et al. 2012) employing the MMF94 force field. Partial charges were assigned, and structures were saved in appropriate formats for docking. The protein–ligand docking studies were performed using AutoDock Vina (Trott and Olson 2010) tool of the UCSF-Chimera v 1.13.1 software (Pettersen et al. 2004). X-ray crystal structure of dopamine receptors D2R (PDB ID: 6CM4, resolution 2.8 Å, co-crystallized with risperidone), D3R (PDB ID: 3PBL, resolution 2.8 Å, co-crystallized with eticlopride), and D4R (PDB ID: 5WIU, resolution 1.9 Å, co-crystallized with nemonapride) were retrieved from the RCSB Protein Data Bank server (https://www.rcsb.org/). Prior to docking, all crystallographic water molecules, co-crystallized ligands, and non-protein structures were removed. Missing hydrogen atoms were added, and protonation states of amino acid residues were assigned according to physiological pH (7.4) in UCSF Chimera. Polar hydrogen and Kollman charges were added using UCSF Chimera (https://www.cgl.ucsf.edu/chimera/). The binding site was identified by the co-crystallized ligand position with a standard grid box (25 × 25 × 25 Å) to ensure full coverage of the ligand-binding pocket. An exhaustiveness value of 8 was used, and for each compound, 10 binding poses were generated. The best binding conformation was selected based on the lowest docking score (kcal/mol) and visual inspection of key interactions with critical residues. The docking protocol was validated by re-docking the co-crystallized ligands into their respective binding sites, yielding RMSD values below 1.5 Å relative to the experimental poses.

#### Molecular dynamics simulations

To understand the structural stability of dopamine receptors as well as of complexes with amathamide G and its derivatives under physiological conditions, these were evaluated using molecular dynamics (MD) simulations using GROMACS 2023.3 software version (Abraham et al. 2015). The systems were built using the CHARMM-GUI web server (Jo et al. 2008), which enabled the proper embedding of the receptors into a lipid bilayer environment. The transmembrane orientation of each receptor was maintained according to the coordinates provided by CHARMM-GUI during membrane insertion. Simulations performed using CHARMM36m force field (Huang et al. 2017) were employed to proteins and lipids; for ligands, parameters were generated using the CHARMM General Force Fiel (CGenFF) (Vanommeslaeghe et al. 2010). Each receptor-ligand complex was inserted into a heterogeneous lipid bilayer composed of cholesterol (CHO, 30%), POPC (45%) and POPE (25%), mimicking a biological relevant membrane environment. The systems were solved using TIP3P water model. Neutralization of the system was carried out by the addition of ions (Na^+^ and Cl^−^) to achieve a final concentration of 0.15 M. The energy minimization process was carried out using the steepest descent algorithm until convergence (< 10 kJ/mol). Equilibration was conducted in two phases: first, an equilibration under the NVT ensemble at 310 K for 5 ns, followed by NPT ensemble at 1 bar and 310 K for 10 ns. During the equilibration stages, positional restraints were applied to the protein atoms to preserve the structural integrity of the receptor while allowing solvent molecules and the lipid bilayer to adapt around the embedded receptor. These restraints were removed prior to the unrestrained production simulations. Temperature was controlled using V-rescale thermostat (Bussi et al. 2007) with a reference temperature of 310 K and a time constant of 0.1 ps. Pressure was maintained using C-rescale barostat (Bernetti and Bussi 2020) with a reference pressure of 1 bar and time constant of 2.0 ps. Long-range electrostatic interactions were treated using the Particle Mesh Ewald (PME) method, and a cut-off distance of 1.0 nm was applied for short-range interactions. Production of MD simulations was conducted for 100 ns with a time step of 2 fs integration time step. All covalent bonds involving hydrogen atoms were constrained using the LINCS algorithm. To analyze the stability of protein and protein–ligand complex systems, several parameters including root mean square deviation (RMSD), root mean square fluctuation (RMSF), solvent accessible surface area (SASA) and hydrogen bonds were calculated throughout the simulations.

#### MM-PBSA and binding energy calculations

The binding free energy of protein–ligand complexes were calculated using gmx-MMPBSA tool from GROMACS (Kumari et al. 2014; Valdés-Tresanco et al. 2021). The MM-PBSA approach was utilized for the binding free energy predictions from MD simulation trajectories in explicit solvent, with three components: complex, receptor and ligand separately. The representation of the binding free energy (ΔG_bind_) of the lead compounds in complex with protein was calculated using the following equation:

$$ \Delta {\mathrm{G}}_{{{\mathrm{bind}}}} = {\text{ G}}_{{{\mathrm{complex}}}} {-} \, \left( {{\mathrm{G}}_{{{\mathrm{protein}}}} + {\text{ G}}_{{{\mathrm{ligand}}}} } \right) $$

The expression can be further expressed as:

$$ \Delta {\mathrm{G}}_{{{\mathrm{bind}}}} = \, \Delta {\mathrm{E}}_{{{\mathrm{MM}}}} + \, \Delta {\mathrm{G}}_{{{\mathrm{solv}}}} {-}{\text{ T}}\Delta {\mathrm{S}} $$

where ΔG_MM_ represents the molecular mechanics energy (van der Waals and electrostatic contributions), ΔG_solv_ corresponds to the solvation free energy (polar and nonpolar terms), and − TΔS accounts for the entropic contribution to binding.

For the MM-PBSA calculations, a total of 5000 evenly spaced frames were extracted from the production phase of the MD simulations (100 ns) to ensure adequate conformational sampling. All calculations were performed at a temperature of 310 K, consistent with physiological conditions. The entropic contributions (-TΔS) to the binding free energy were also included to improve accuracy in MM-PBSA estimations. Therefore, the reported binding energies correspond to the enthalpic/entropic balances of the system.

## Results and discussion

### Chemistry

**Scheme 1:**
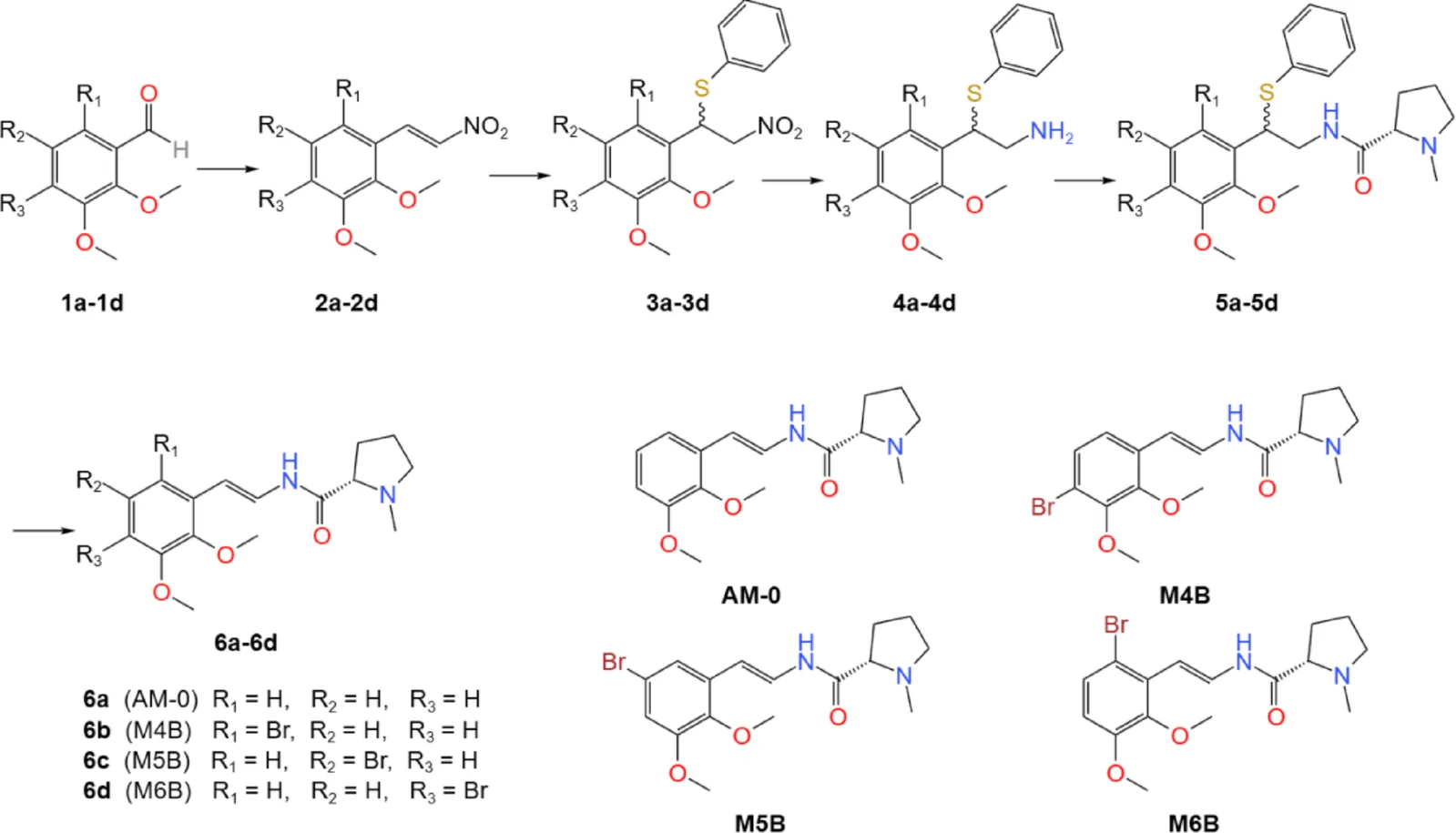
Scheme for synthesis of amathamide G derivatives **6a**–**6d**. **a** AcONH4, CH3NO2, AcOH, reflux **b** PhSH, **c** Zn, HCl, AcOH, **d** N-methtyl-L-proline, HOBt, EDC:HCl, DIPEA, CH2Cl2, **e** I. NaIO4, AcOH/H2O, II. K2CO3, xylene, reflux

**Fig. 2:**
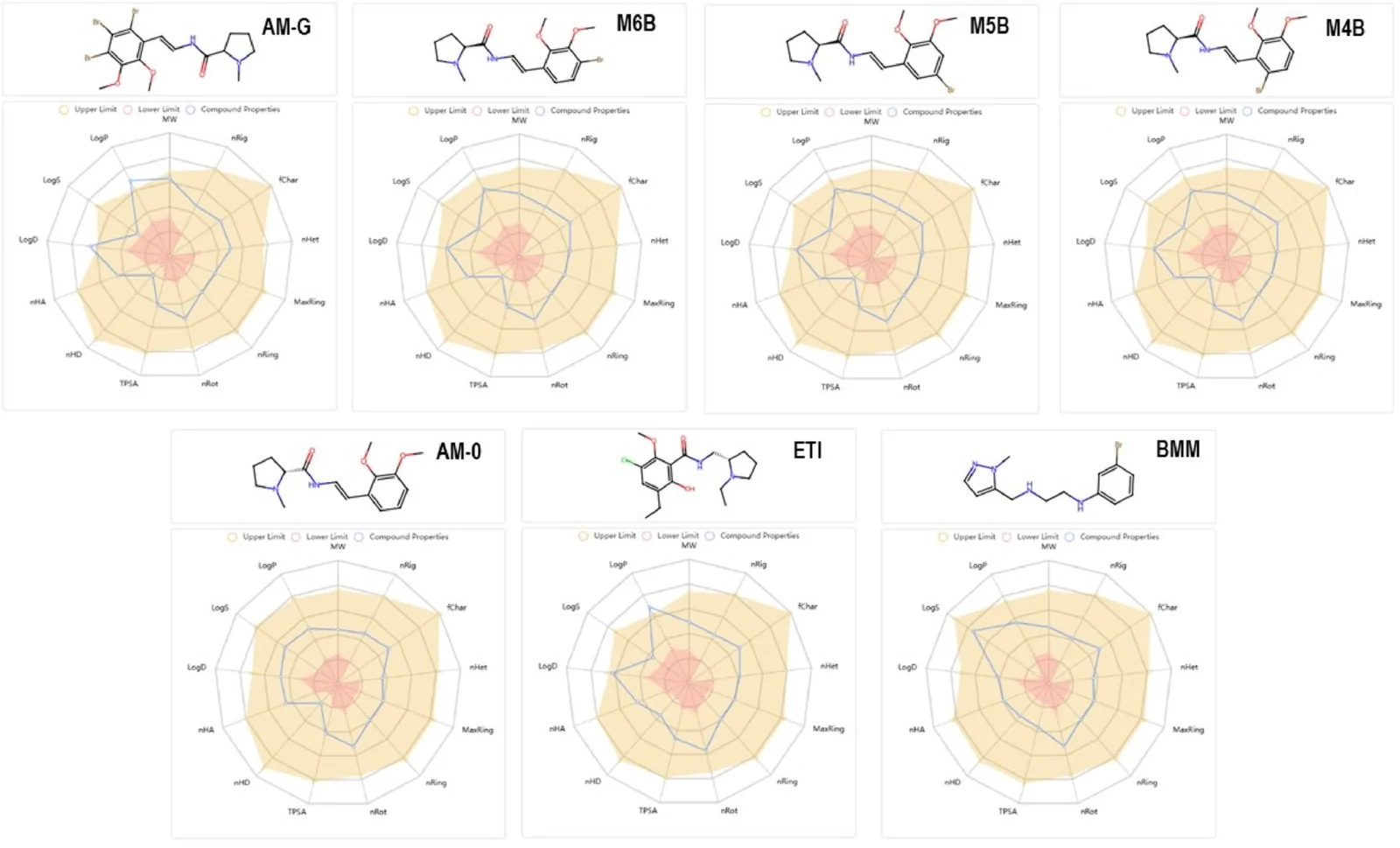
Radar diagram of physicochemical properties of amathamide G, monobrominated derivatives and the reference compounds ETI and BMM. Yellow and red zones indicate the recommended and undesirable limits, respectively, while blue contours represent the computed values. The progressive reduction in bromine substitution from M6B to M4B, and AM-0 led to decreased *logP* and *logD* values, placing all monobrominated derivatives within favorable ranges. For ETI, only the *logP* exceeded the optimal range. Data was retrieved from ADMETLab 2.0 server

The amathamide G derivatives include a non-brominated **6a** (AM-0) and three monobrominated derivatives **6b**–**6d** (M4B, M5B and M6B, respectively) which were obtained as depicted in Scheme 1. Initially, condensation of **1a**–**1d** with nitromethane in the presence of ammonium acetate afforded the nitroolefins **2a**–**2d**. Subsequent Michael addition of thiophenol to each nitroolefin yielded the adducts **3a**–**3d**. Reduction of the nitro group using Zn under acidic conditions provided the amines **4a**–**4d**. Acylation of these amines with N-methyl-L-proline in the presence of EDC/HOBt afforded the intermediates **5a**–**5d**. Finally, elimination of thiophenol via oxidation with NaIO_4_ to the corresponding sulfoxides followed by refluxing gave the four desired enamides **6a**–**6d** (Fig. 2) (Ramírez-Osuna et al. 2011; Blumberg and Martin 2018; Aguiar et al. 2022). Full characterization details, including ^1^H and ^13^C NMR and EI-MS are provided in the Supplementary information.

Importantly, this series was rationally designed to systematically evaluate the impact of bromination on the aromatic ring, generating a set of derivatives with modifying halogen substitution (AM-0 to M6B). In contrast to previously reported amathamide A derivatives, which have primarily focused on structural modifications of the backbone (Ramírez-Osuna et al. 2005), the present study explores dehalogenation as a strategy to modulate physicochemical and pharmacological properties.

### Computational studies

#### Physicochemical properties

As a first stage of analysis, amathamide G and its monobrominated derivatives, along to the compound eticlopride (ETI), a standard D3R inhibitor (Chien et al. 1979) and N´(3-bromophenyl)-N-((2-methylpyrazol3-yl)methyl)ethane−1,2-diamine (ZINC97048126; BMM), a reference compound reported as D4R antagonist (Wang et al. 1979), were evaluated using the ADMETLab2.0 platform (Fu et al. 2024). This server provides a multi-level calculation of molecular descriptors, properties and biological parameters of compounds, from which we started with the analysis of thirteen computed physicochemical properties.

As can be seen in Scheme 1, the successive debromination of the amathamide G scaffold (from the tribrominated AM-G to the non-brominated AM-0) resulted in modifications in two properties related to solubility, namely *logP* and *logD*. The first corresponds to the partition coefficient octanol/water and the latter to the liposolubility at physiological pH (7.4). In the case of AM-G, these parameters were above the upper limit; however, upon the loss of two and three bromine atoms, these parameters were diminished, indicating a predicted reduction in molecule's solubility in lipophilic media, including physiological conditions (pH 7). This could have important implications for an enhanced solubility of non-brominated derivative AM-0 in aqueous environments such as interstitial fluid, gastric fluid and plasma while retaining a moderate lipophilicity (yellow zones in Fig. 2) which is commonly associated to good permeation across biological membranes (Latif 2024). Likewise, a *logP* value of 1–3 is considered optimal for brain penetration (Janicka et al. 2020) and this range was met too in non-brominated derivative. Accordingly, all the computed properties of AM-0 fit nicely within minimal and maximal values (see Supplementary information, Table S1).

For the standard compound ETI, only the *logP* value was above the upper limit, likely due to the presence of a chlorine atom that is nevertheless smaller than bromine; likewise, its absence could diminish in the same manner the *logP* and *logD* parameters of this compound (data not shown). This observation further supports the notion that the removal of one or multiple halogen atoms favorably modifies the liposolubility of the amathamide G derivatives. In the case of the reference compound BMM, all parameters fell within optimal ranges and only its aqueous solubility index *log S* was higher than that of all remainder compounds.

On the basis of structural comparisons, there are common features among derivatives, ETI and BMM that explain their relatively similar physicochemical profiles. One is the presence of a benzene ring and a saturated or unsaturated pentacyclic ring, both joint by a partially saturated aliphatic linker 3–5 backbone atoms long; the second is that a halogen atom (Br, Cl) is attached to the benzene ring. In this context, it is well recognized that adding halogens in chemical structures has an impact not only on the physicochemical properties of a given compound (i.e. solubility, stability) but on its electronic distribution, and hence ability to interact with specific targets as reported in microbial and cancer cells (Barreto et al. 2025; Diederich et al. 2025). Interestingly, there is evidence suggesting a role of halogenation on the selectivity of alkaloids towards DRs. For instance, halogenation with Cl, Br or I at the position 3 of the aporphine alkaloid boldine enhances their selectivity for D1R-like over D2R-like entities (Asencio et al. 2005). In other study, 1-phenylbenzazepines with mono- or di-bromination/chlorination at the ortho position of ring C modify their patterns of interaction with D1R, D2R and D5R compared to their non-halogenated counterparts (Giri et al. 2020). Moreover, the bromination at C12 of tetrahydroprotoberberine alkaloids tends to increase the selectivity towards D1R (Madapa et al. 2017). These observations highlight a potential role of halogenation/dehalogenation of amathamide G derivatives, particularly at the benzene moiety, on their potential interactions with DRs.

#### Medicinal chemistry evaluations

In these analyses, combinations of relevant properties of compounds including MW, *logP*, *logD*, TPSA, nHA, nHD and nRot are used as criteria for approval or rejection of a given candidate as “lead drug” or “drug-like” with potential pharmacological effects in the human body. Platforms intended for this evaluation commonly include the Lipinsky´s Rule of Five (RO5), Pfizer Rule, Glaxo-Smith Kline (GSK) Rule and the Golden Triangle Rule. Two molecular descriptors describing the likeliness of a desirable pharmacology (QED) and the possibility for further molecular evolution or novelty (MCE−18) were also included (Table 1).

**Table 1:** Medicinal Chemistry evaluation of amathamide G (AM-G) and its derivatives (AM-0, M6B, M5B and M4B), compared with the standard (ETI) and reference (BMM) compounds

Parameter	AM-G	M6B	M5B	M4B	AM-0	ETI	BMM
QED^1^	0.58(-)	0.87( +)	0.86( +)	0.86( +)	0.89( +)	0.83( +)	0.80( +)
MCE-18^2^	50.21( +)	43.52( −)	43.52( −)	43.52( −)	40.17( +)	46.14( +)	10.0( −)
Lipinski rule^3^	( +)	( +)	( +)	( +)	( +)	( +)	( +)
Pfizer rule^4^	( −)	( +)	( +)	( +)	( +)	( −)	( +)
GSK rule^5^	( −)	( +)	( +)	( +)	( +)	( +)	( +)
Golden Triangle rule^6^	( −)	( +)	( +)	( +)	( +)	( +)	( +)

Decision: approved ( +), rejected ( −) and moderate risk/safeness ( ±)

^1^A measure of drug-likeness based on the concept of desirability: Attractive: > 0.67; unattractive: 0.49~0.67; too complex: < 0.34

^2^MCE-18 stands for medicinal chemistry evolution. MCE-18 ≥ 45 is considered suitable value

^3^ MW < 500; *logP* < 5; Hacc < 10; Hdon < 5. If two properties are out of range, a poor absorption or permeability is possible, one is acceptable

^4^*logP* > 3; TPSA < 75. Compounds with a high *logP* (> 3) and low TPSA (< 75) are likely to be toxic

^5^MW < 400; *logP* < 4. Compounds satisfying the GSK rule may have a more favorable ADMET profile

^6^200 < MW < 50; − 2 < *logD* < 5. Compounds satisfying the Golden Triangle rule may have a more favorable ADMET profile

In these six evaluations, AM-G was the only alkaloid rejected by QED and Golden Triangle Rule, whereas all derivatives were rejected by MCE-18 (i.e. they display lower sp3 or 3D novelty, being this reasonable as these are derivatives of the already known compound AM-G). Notably, all compounds were approved by the RO5, which has more relaxed criteria for acceptance (MW < 600, *logP* < 5) (Lipinski et al. 2001), while AM-G was rejected by all remainder rules hence derivatives approved all four rules. Meanwhile, ETI approved five out of six criteria with the exception of the Pfizer rule, due to its MW > 500 and *logP* > 3 (Supplementary information, Table S1) and BMM was rejected only by MCE-18 but by a much broad margin (index 10 of 45) than monobrominated (43 of 45) or non-brominated (40 of 45) derivatives. In a general landscape and in agreement with physicochemical properties, medicinal chemistry evaluations mostly favored the derivatives over AM-G as more stable molecules with a potentially safer pharmacological profile. However, to obtain a more comprehensive prediction of the drug likeness of these alkaloids for application in CNS, it was necessary to conduct a pharmacokinetic predictive evaluation.

#### Pharmacokinetic predictions

As a third evaluation of AM-G and its derivatives, their ADMET (absorption, distribution, metabolism, excretion and toxicity) profiles were predicted by using the ADMETlab 2.0 web platform, considering key parameters for each of these processes (Table 2).

**Table 2:** Predicted pharmacokinetic parameters of amathamide G (AM-G) and its derivatives (AM-0, M6B, M5B and M4B), compared with the standard (ETI) and reference (BMM)

	Parameter	AM-G	M6B	M5B	M4B	AM-0	ETI	BMM
A	HIA (*P*)	0.41( ±)	0.01( +)	0.01( +)	0.01( +)	0.01( +)	0.01( +)	0.01( +)
	BBBp (*P*)*	0.98( +)	0.99( +)	0.98( +)	0.99( +)	0.95( +)	0.96( +)	0.96( +)
D	Volume of distribution (L/Kg)	0.95( +)	1.13( +)	1.18( +)	1.12( +)	1.21( +)	1.40( +)	2.99( +)
	PPB(%)	96.31( −)	75.72( +)	63.22( +)	73.93( +)	25.09( +)	90.24( +)	87.01( +)
M	CYP1A Inhibitor (*P*)** Substrate (*P*)	0.722 **0.961**	**0.939** **0.933**	**0.928** **0.914**	**0.921** **0.940**	0.618 **0.944**	0.737 0.249	0.683 0.723
	CYP2D6 Inhibitor (*P*)** Substrate (*P*)	0.486 **0.877**	0.420 **0.911**	0.669 **0.913**	0.613 **0.917**	0.473 **0.922**	**0.854** 0.402	0.748 **0.884**
	CYP3A4 Inhibitor (*P*)** Substrate (*P*)	0.766 **0.910**	0.589 **0.917**	0.592 **0.908**	0.632 **0.912**	0.250 **0.869**	0.118 0.258	0.254 0.218
E	Clearance (mL/min/kg)	1.52( −)	2.27( −)	5.15( +)	6.88( +)	8.62( +)	4.12( −)	6.60( +)
	Half-life (T1/2, h)	0.408	0.775	0.830	0.776	0.876	0.181	0.707
T	Rat oral toxicity (*P*)	0.89( −)	0.85( −)	0.60( ±)	0.74( ±)	0.49( ±)	0.13( +)	0.87( −)
	DILI (*P*)	0.36( ±)	0.26( +)	0.22( +)	0.56( ±)	0.32( ±)	0.18( +)	0.22( +)
	Carcinogenicity	0.06( +)	0.51( ±)	0.36( ±)	0.49( ±)	0.42( ±)	0.38( ±)	0.13( +)

Decision: approved ( +), rejected ( −) and moderate risk/safeness ( ±)

HAI, Human intestinal absorption; BBBp, Blood–brain barrier penetration; PPB, Plasma protein binding; DILI, Drug-induced liver injury; *P*, probability (0–1)

*For compounds intended to act at the CNS (i.e. DRs), high *P* value in this parameter is considered as approved

**Values in bold indicate a high probability to interact with the referred cytochrome

At the level of absorption (A), AM-G exhibited the lowest probability (*P* = 0.59) to be absorbed by ≥ 30% (threshold), which could be derived from its non-optimal, high *logP* value (3.8). Meanwhile, all monobrominated derivatives, ETI and BMM exhibited a good probability to permeate the brain-blood barrier (> 95%) by ≥ 30%, a favorable feature considering that DRs are localized at CNS and also are consistent with their *logP* values (range 2–3; Supplementary information, Table 1). In terms of distribution (D), all compounds have a good volume of distribution; however, AM-G and ETI exhibited a high proportion (> 90%) of plasma protein that would limit their CNS bioavailability, AM-0 displayed a much lower proportion (25%) and the monobrominated derivatives were predicted to have a more balance binding proportion (63–75%). BMM had a higher but still satisfactory ability to bind plasmatic proteins (87%). Regarding metabolism (M) of drugs by the cytochrome P450 (CYP) heme-containing enzyme superfamily (comprising 57 CYP in humans) (Guengerich 2008), where the CYP1-3 groups are the most prominent drug-converting members, it is worth of note that all monobrominated derivatives had a significant possibility (*P* > 0.85) to interact as substrates of CYP1A2, CYP2D6 and CYP3A4, which participate in xenobiotic and steroid metabolism through a predominating monooxygenase activity (Nelson 2018). Interestingly, only the monobrominated derivatives have a high probability (*P* > 0.90) to be CYP1A2 inhibitors. ETI only displays significant probability (*P* > 0.85) to inhibit CYP2D6 and BMM to be a CYP2D6 substrate (*P* > 0.85). These predictions suggest that monobrominated derivatives and BMM are more likely susceptible to metabolization than the standard drug ETI. Here, the romine substituents do not have apparent effect.

When excretion (E) parameters were analyzed, a difference was noted among the amathamide G derivatives. Specifically, only M6B and AM-G itself had a low clearance rate (< 2.5 mL/min/kg) compared to remaining derivatives, which showed higher clearance rates (> 5 mL/min/kg). In addition, half-lives were longer for derivatives (> 0.75 h.) than for AM-G (0.40 h.), suggesting an inverse correlation between half-life and the grade degree of bromination of derivatives. ETI was the compound with the shortest half-life (0.18 h.) besides a low clearance (4.1 mL/min/kg) and BMM displayed good excretion parameters. Finally, the values retrieved for potential toxicity (T) shed interesting insights on the pharmacological profile of monobrominated derivatives compared to ETI and BMM: (a) only ETI showed a low risk of oral acute toxicity in rats, followed by the M5B, M4B and AM-0 while AM-G and M6B had a high risk, denoting a lower risk of toxicity upon diminishing bromine substituents. In this parameter, BMM exhibited a high risk, (b) the risk of drug-induced liver injury (DILI) is relatively low with monobrominated derivatives and for M6B and M5B it is even comparable to that of ETI and BMM, and (c) in contrast to other trends, decreasing the degree of bromination from AM-G resulted in a moderate to higher predicted carcinogenicity risk, comparable to that of ETI (*P* > 0.35). In a similar way to AM-G, BMM displayed a very low risk of carcinogenicity.

In a general view, all amathamide G derivatives are predicted to reach the CNS if administered by either enteral or parenteral routes, have high probabilities of metabolization by CYPs 1–3 (e.g. monooxigenation), low to moderate hepatotoxic potential and medium risk of carcinogenicity. Nonetheless, the presence of less bromine substituents might favorably modify some pharmacokinetic parameters such as balancing their carrying by plasmatic proteins, accelerating clearance and extending half-life. All these features would afford monobrominated derivatives an enhanced bioavailability at target organs or tissues and a satisfactory pharmacological profile.

In the light of these analyses, it could be assumed that monobrominated derivatives exhibit slightly distinct set of physicochemical properties and a medicinal chemistry profile broadly comparable to those of the reference compounds ETI and BMM. Notably, several pharmacokinetic parameters —including plasma protein binding, clearance, and half-life—were predicted to improve relative to AM-G. However, these potential advantages should be interpreted alongside the predicted toxicity liabilities, including oral toxicity, DILI risk, carcinogenicity alerts, and possible CYP-mediated interactions observed for several derivatives. It is important to emphasize that these in silico predictions, derived from machine-learning and QSAR-based models, should be interpreted as early safety indicators for guiding lead optimization, rather than as definitive toxicological assessments. Consequently, while the monobrominated derivatives emerged as promising candidates for further investigation, their therapeutic potential remains to be established through experimental pharmacological and toxicological studies. Given their structural similarity to reference compounds ETI and BMM, we next tested the hypothesis that monobrominated derivatives may CNS targets, specifically dopamine receptors.

#### Target prediction

To identify potential targets of synthesized compounds, the structures of AM-G and its monobrominated derivatives were uploaded to the SwissTargetPrediction database (http://www.swisstargetprediction.ch/). The website performs target fishing using ligand-based target prediction methods that are based on the molecular similarity principle (Gfeller et al. 2014). The initial predictions pointed to multiple targets, predominantly members of the GPCRs family, followed by kinases. To refine the analysis, we employed the SwissSimilarity platform (Zoete et al. 2016), which implements a 3D similarity search with the ElectroShape method (Armstrong et al. 2010), an approach that integrates electrostatic and shape-based information within a unified framework. The screening process of drugs structurally similar to AM-G and its derivatives, identified the standard ETI as well as Fallypride, Remoxipride and Sulpiride, compounds used in the treatment of schizophrenia that act on dopamine receptors of the D2-like family (Wong and Van Tol 2003). Specifically, ETI and Fallypride are strong antagonistic of D2R and D3R (Martelle and Nader 2008; Chien et al. 1979; Vyas et al. 2018), likewise Remoxipride (Nadal 2001) and Sulpiride (Caley and Weber 1995) are D2R antagonistic but also exhibit inhibitory activity against D3R and D4R. Interestingly, all of the structurally related compounds retrieved by SwissSimilarity are clinically relevant dopaminergic agents that recognize members of the D2-like receptor family. The convergence of two independent ligand-based approaches strengthens the rationale for selecting D2-like dopamine receptors for subsequent structural analyses. Although ligand-based target prediction cannot establish biological activity or receptor specificity per se, the concordance between these complementary approaches provides a biologically plausible basis for focusing on D2R, D3R, and D4R in the ensuing the subsequent docking and molecular dynamics studies. Evaluating the three receptor subtypes enabled a comparative computational assessment of interaction trends across the D2-like receptor family, rather than restricting the analysis to a single receptor subtype.

### Molecular docking studies

Docking studies were performing using AM-G and its monobrominated derivatives against dopamine receptors D2R (PDB: 6CM4), D3R (PDB: 3PBL) and D4R (PDB: 5WIU). Dopamine receptors possess a ligand-binding pocket located in the upper half of their transmembrane domains (TMS), which is composed of orthosteric binding pocket (OBP), and extended binding pocket (EBP). All compounds were docked into a cubic grid box of 25×25x25 Å, centered on the position of the co-crystallized ligands, which encompassed both the OBP and EBP regions.

Additionally, the co-crystallized ligands of each receptor (risperidone for D2R, ETI for D3R, and nemonapride for D4R), were considered as structural references to validate the docking protocol and provide a benchmark for binding interactions. A re-docking approach was performed using the crystallographic structure and their respective ligands (with RMSD < 1.5 Å), allowing a direct comparison between predicted and experimental binding poses (Supplementary information, Fig. S48). The results indicated that all compounds exhibited favorable docking score values and occupied the canonical binding pocket of the receptors, showing interaction patterns consistent with those observed for known antagonists (Table 3).

**Table 3:** Binding score (kcal/mol) of AM-G, its derivatives, and reference compounds against dopamine receptors

Compounds	D2R	D3R	D4R
AM-G	− 7.6 ± 0.3	− 7.0 ± 0.1	− 8.2 ± 0.5
M4B	− 7.5 ± 0.1	− 7.5 ± 0.1	− 8.4 ± 0.1
M5B	− 7.5 ± 0.1	− 7.4 ± 0.1	− 8.5 ± 0.2
M6B	− 7.6 ± 0.1	− 7.2 ± 0.1	− 8.6 ± 0.9
AM-0	− 8.3 ± 0.2	− 7.2 ± 0.2	− 7.9 ± 0.1
ETI	− 4.5 ± 2.0	− 3.9 ± 1.9	− 7.1 ± 1.2
BMM	− 7.1 ± 0.2	− 7.0 ± 0.2	− 7.4 ± 0.1

**Fig. 3:**
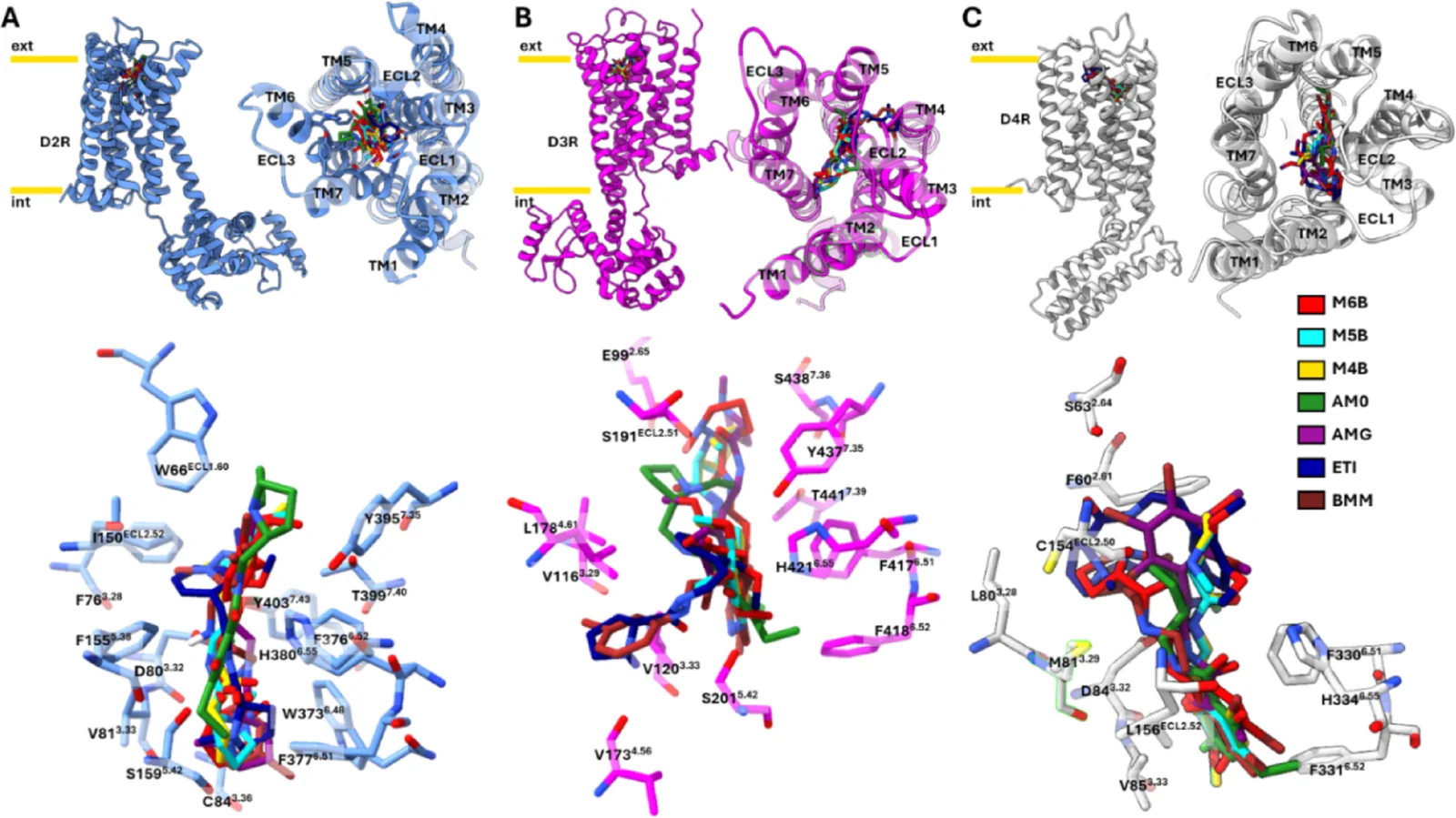
Docking positions (upper, whole proteins) and molecular interactions (lower, interacting amino acids) of amathamide G and its monobrominated analogs at **A** D2R, **B** D3R and **C** D4R

In the D2R receptors model (Fig. 3A), docking studies revealed that AM-G and monobrominated derivatives formed a hydrogen bond and an ionic interaction with the conserved D80^3.32^ residue (superscripts are according to the Ballesteros-Weinstein scheme (Ballesteros and Weinstein 1995)). Furthermore, monobrominated derivatives also form hydrophobic contacts with the residue I150 ^ECL2.52^ and less with V81^3.33^, F376^6.51^, F377^6.52^ and T399^7.40^. AM-0 and ETI did not form a hydrogen bond with D80^3.32^, but they had hydrophobic contacts with F376^6.51^ and F377^6.52^. Finally, BMM formed a hydrogen bond and ionic interaction with D80^3.32^, and hydrophobic contacts with I150^ECL2.52^. The generation of hydrogen bonds at residue D80^3.32^ is relevant for the interaction of agonist and antagonist compounds (Wang et al. 2018; Fan et al. 2020). On the other hand, it has been observed that F376^6.51^ and I150 ^ECL2.52^ are essential for the interaction of the antagonist spiperone as mutating them drastically reduces the affinity (Cho et al. 1995; Im et al. 2020).

In the case of D3R (Fig. 3B), none of the compounds interacted with residue D^3.32^, albeit the monobrominated compounds interacted with E99^2.65^, one of the important interaction residues of the antagonists. Interestingly, ETI and monobrominated interact with two aromatic residues that are part of a subpocket F417^6.51^ and F418^6.52^, in addition to forming an interaction with S201^5.42^ along with BMM, a characteristic feature of the agonist cathecolamine (Braden and Nichols 2007; Ferruz et al. 2018).

About the D4R receptor model (Fig. 3C), docking studies revealed that AM-G, M4B and ETI interact with the same residues, displayed a hydrogen bond with D84^3.32^, and hydrophobic contacts with V85^3.33^ and F330^6.51^. In a slightly different manner, BMM forms hydrophobic contacts with M81^3.29^ F330^6.51^ and F331^6.52^. M5B, M6B and AM-0 did not form a hydrogen bond with D84^3.32^ but did with hydrophobic contacts with V85^3.33^ and L156^4.43^.

Overall, the interaction profiles of the monobrominated derivatives shared several features with those reported for reference dopaminergic ligands, including contacts with conserved residues involved in ligand recognition within D2-like receptors (Xu et al. 2023). However, these similarities should be interpreted cautiously, as docking analyses provide structural hypotheses regarding receptor–ligand interactions but do not establish pharmacological selectivity or distinguish between agonist and antagonist behavior. Experimental receptor-binding and functional studies will be required to validate the pharmacological significance of these predicted interactions.

Molecular docking results suggest that AM-G and its derivatives exhibit favorable binding scores for D2R and D4R, ranging from − 7.1 to − 8.3 kcal/mol. However, these scores should be interpreted with caution, as AutoDock Vina has an estimated error of approximately ±2.5 kcal/mol (Trott and Olson 2010). For D2R, AM-0 exhibited the most favorable docking score (− 8.3 kcal/mol), followed by AM-G (− 7.6 kcal/mol), and monobrominated derivatives (− 7.45 to − 7.66 kcal/mol). BMM had slightly lower affinity (− 7.1 kcal/mol), while ETI was the one with the lowest docking score (− 5.2 kcal/mol). In D3R, ETI had the worst affinity values (− 3.9 kcal/mol) compared to monobrominated derivatives (− 7.2 to − 7.5 kcal/mol), AM-G (− 7.0 kcal/mol) and AM-0 (− 7.2 kcal/mol). For D4R, the monobrominated derivatives showed highest values (− 8.4 to − 8.6 kcal/mol) whereas BMM (− 7.4 kcal/mol) and ETI (− 7.1 kcal/mol) showed the lowest ones. Interestingly, ETI and BMM showed lowest predicted scores across all three receptors compared to some of the monobrominated derivatives, despite their known antagonist profile for D3R and D4R, respectively (Chien et al. 1979; Wang et al. 1979).

Overall, the docking results suggest that AM-G and its derivatives are capable of interacting with the canonical binding site of D2-like dopamine receptors and establishing interactions with residues implicated in ligand recognition. Although some derivatives exhibited more favorable docking scores toward D4R relative to D2R and D3R, these differences should be interpreted cautiously given the inherent limitations and uncertainty associated with docking scoring functions. Therefore, the observed trends are more appropriately considered as qualitative indicators useful for prioritizing compounds for subsequent analyses rather than evidence of true receptor selectivity or binding affinity. Because docking provides a static representation of receptor–ligand interactions and does not fully account for protein flexibility or solvent effects, molecular dynamics simulations were subsequently performed to further evaluate the stability and dynamic behavior of the predicted complexes.

### Molecular dynamics simulations

The dynamic stability of the docked complex and the flexibility of the dopamine receptors were assessed using the MD simulations. MD simulations are widely used to investigate the dynamic behavior of proteins or protein–ligand complexes. In this study, the stability of complex formed between AM-G and its derivatives with D2R, D3R and D4R, all these embedded into membrane bilayers with typical biologic composition, were evaluated over a 100 ns simulation using GROMACS software. The MD simulation trajectory was used to compute several parameters, including the root-mean-square deviation (RMSD) of protein Cα atoms, root-mean-square fluctuation (RMSF) of all systems, solvent accessible surface area (SASA) and hydrogen bonds, to assess their stability and dynamic behavior.

**Fig. 4:**
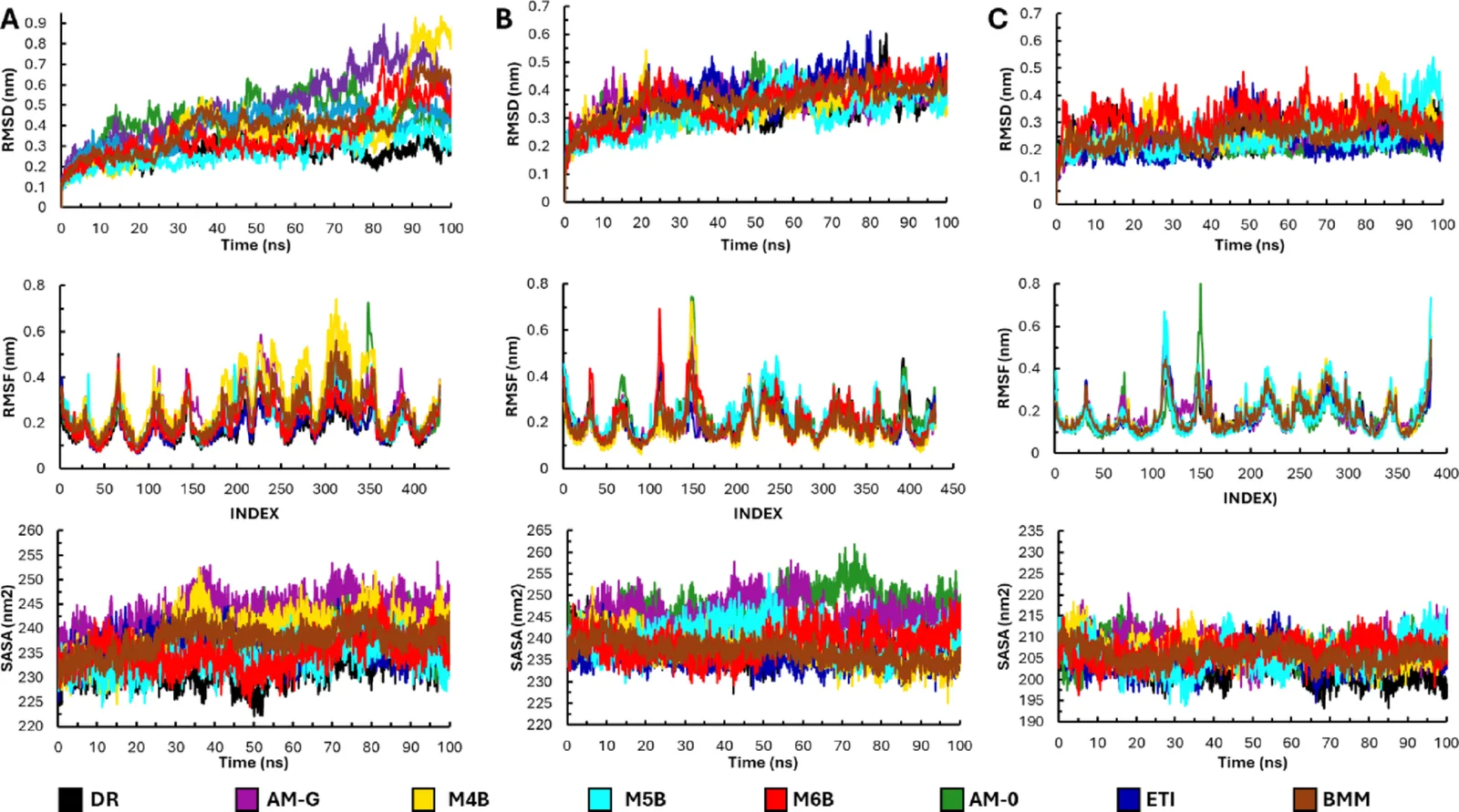
Molecular dynamics simulation of the protein–ligand for 100 ns. The RMSD, RMSF and SASA graphs of the compounds with **A** D2R, **B** D3R and **C** D4R embedded in membrane bilayers are shown

Firstly, RMSD evaluation provides an estimate of the overall stability and dynamic behavior of the protein–ligand complex. RMSD plots of D2R, D3R and D4R complexes with AM-G and its derivatives are shown in Fig. 4. Slightly higher RMSD values observed in complexes may reflect local conformational adjustments that are commonly observed in GPCR systems embedded within membrane environments. This analysis indicated that most complexes reached equilibrium after initial fluctuations, maintaining values below ~0.5 nm, suggesting overall structural stability. For D2R complexes, RMSD values ranged from 0.22 to 0.9 nm with a mean of 0.43 nm (Fig. 4A, top), indicating overall stability. Most complexes remain stable throughout the simulation; only AM-G and M4B show a slight increase (0.50 and 0.52 nm, respectively). D3R and D4R complexes (Fig. 4B, C, top) showed greater stability compared to D2R, with values from 0.14 to 0.56 nm and an average around 0.30 nm. Similarly, the RMSF in D3R and D4R (0.17–0.20 nm) showed fewer fluctuations compared to D2R (∼0.23 nm). Specifically, the D2R-M4B complex shows the greatest fluctuations in the intracellular domain region (0.31 nm). These findings suggest that most residues maintained relatively limited fluctuations throughout the simulations, indicating preservation of the overall receptor architecture in the presence of the ligands.

The changes in structural features of complexes were also analyzed by computing the SASA parameter (Fig. 4). In D2R complexes, the compounds increased slightly their solvent-accessible surface (∼237.96 nm^2^) with respect to protein alone (232.54 ± 3.16 nm^2^). In D3R complexes, the compounds increased slightly (242.15 nm^2^) with respect to the protein alone (241.79 ± 5.62 nm^2^), with the exception of ETI and M4B which slightly reduced to 237.01 and 236.18 nm^2^ respectively. Finally, in D4R complexes and similar to D2R, a slight increase was afforded with the compounds (207.83) compared to protein alone (204.33 nm^2^).

Collectively, these results indicate that the receptor–ligand complexes remained structurally stable throughout the simulation time and did not undergo major conformational disruptions after equilibration. The observed dynamic behavior supports the plausibility of the predicted binding modes obtained from docking analyses. Nevertheless, these findings should be interpreted as evidence of the stability of the simulated complexes rather than as direct indicators of receptor affinity, selectivity, or pharmacological activity.

**Fig. 5:**
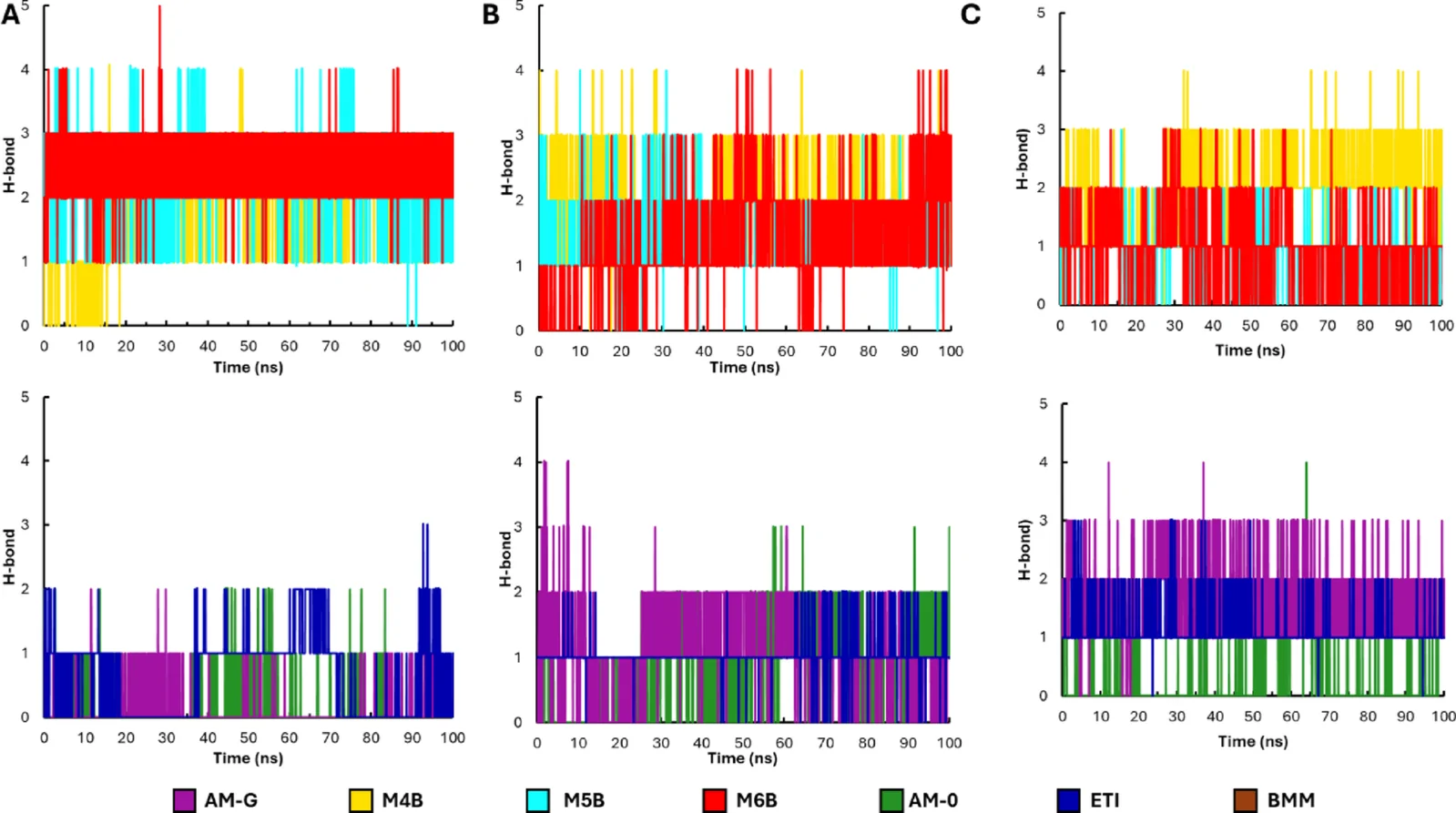
Hydrogen bonds formed during the simulation of compounds with **A** D2R, **B** D3R and **C** D4R embedded in membrane bilayers

Hydrogen bonds are key determinants of protein–ligand complex stability and flexibility. Hydrogen bond analysis revealed moderate interactions across systems; however, these bonds were not the primary contributors to binding stability. Results with D2R complexes (Fig. 5A) showed that The monobrominated derivatives exhibited higher hydrogen bonding rates, with an overall average of ~2.2 hydrogen bonds per frame throughout the simulation, most of which were two- and three-bonds. Specifically, M6B showed an average of 2.4 ± 0.5 hydrogen bonds per frame. In contrast, ETI, AM-0 and AM-G complex exhibited the lowest average of hydrogen bonds formed (0.56, 0.12 and 0.04, respectively). In D3R complexes (Fig. 5B), the AM-G and monobrominated compounds had a slight overall increase in hydrogen bonds per frame (~ 1.6) compared with ETI and AM-0 (~ 1.03). Particularly, M4B showed an average of 2.3 ± 0.6 hydrogen bonds per frame. In D4R complexes (Fig. 5C), similarly to what was observed in D3R, M4B displayed the highest number of hydrogen bonds (1.9 per frame), while M5B, M6B, ETI, and AM-G presented an average of 1.1 hydrogen bonds per frame. Finally, AM-0 presented the lowest number, with an average of 0.3 hydrogen bonds per frame. These results indicate that hydrogen bonding is not the dominant interaction stabilizing this complex; furthermore, AM-G and the monobrominated derivatives were capable of forming stable interactions with the three receptors, although not necessarily exceeding those observed for the reference compound ETI.

Taken together, these findings indicate that AM-G and its monobrominated derivatives are capable of maintaining persistent intermolecular interaction within the simulated receptor environments, supporting the structural plausibility of the predicted binding modes. However, the presence and frequency of hydrogen bonds should not be interpreted as direct indicators of receptor affinity, selectivity, or functional activity, which will require experimental validation.

### Binding free energy calculations

The trajectories obtained from MD simulations were also used to estimate the binding free energy (ΔG_bind_) using the MM-PBSA method. These calculations are considered more accurate than the scoring functions of most docking programs for predicting the binding free energy of compounds (Wang et al. 2019). According to the results obtained and presented in Table 4, variations in the binding energy values were observed among the evaluated compounds across the three DRs. Overall, the monobrominated derivatives (M6B, M5B and M4B) and AM-G exhibited the most favorable binding values for the three receptors.

Energy decomposition analysis revealed that van der Waals energy (ΔE_vdw_) was the major contributor to the total binding, followed the electrostatic energy (ΔE_ele_), while non-polar solvation energy (ΔE_np_) contributed favorably but at a lesser extent. In contrast, the polar energy (ΔE_pol_) was generally unfavored. These results suggest that hydrophobic interactions play a dominant role in stabilizing ligand binding within the receptor pocket, suggest that hydrogen bonding plays a secondary role.

In D2R, The AM-G and monobrominated derivatives exhibited more favorable estimated binding free energies than AM-0 (− 9.13 kcal/mol), suggesting that bromination may contribute to the stabilization of the simulated receptor-ligand complexes. Furthermore, there were slight changes in affinity according to the position of the bromine: M6B > M4B > M5B. The presence of a single bromine improves estimated binding free energy, but not the presence of all three bromines, as in AM-G (− 22.64 kcal/mol). These values were consistent with stable hydrogen bonding profiles and overall structural stability observed during the simulations. On the other hand, ETI had a mean affinity value (− 11.64 kcal/mol), suggesting weaker binding affinity compared to other compounds, which supports the notion that these compounds warrant further investigation as potential D2R-interacting molecules.

In contrast, for D3R, ETI was one of the compounds that yielded one of the best scores (− 30.48 kcal/mol), consistent with its known antagonistic activity towards D3R (Wang et al. 1979). Similarly, the monobrominated alkaloids showed affinity comparable to ETI. Although the derivatives have similar affinity values, they can be ordered by affinity as follows: M6B > M4B > AM-0 > M5B > AM-G. Interestingly, AM-0 showed a better affinity for D3R (− 26.72 kcal/mol) compared to D2R, which may indicate a preferential interaction trend toward D3R within the context of the computational models employed.

For D4R, ETI exhibited a similar affinity to that observed for D3R, as did M4B and M5B. Interestingly, M6B showed a half-lower affinity (− 16.22 kcal/mol), indicating that bromination at position 6 adversely affects stability and binding to D4R. On the other hand, the absence of bromination (AM-0) and tribromination (AM-G) affects receptor affinity. These observations indicate that M4B and M5B exhibited comparatively more favorable MM-PBSA estimates within the D4R systems analyzed, providing a rationale for prioritizing these compounds for future experimental characterization.

Importantly, the reference compounds ETI and BMM exhibited favorable binding free energy values consistent with their known activity profiles, supporting the reliability of the simulation setup. Notably, differences in hydrogen bond formation across systems indicate that binding stability depends not only on hydrogen bonding, but also on hydrophobic and electrostatic interactions. Overall, these results suggest that AM-G and its monobrominated derivatives can maintain stable receptor-ligand complexes under the computational conditions evaluated.

Overall, the MM-PBSA analyses identified differences in the predicted stability of receptor–ligand complexes across the D2-like receptor family. Monobrominated derivatives frequently exhibited favorable binding free energy estimates relative to the reference compounds, whereas M4B and M5B displayed particularly favorable profiles within the D4R simulations. Likewise, AM-0 showed comparatively more favorable MM-PBSA estimates in D3R than in D2R or D4R.However, these findings should be interpreted comparatively rather than as absolute binding affinities and should be alongside structural and dynamic analyses.

In summary, MD simulations demonstrated that most protein–ligand complexes remained structurally stable throughout the 100 ns trajectories and provided valuable mechanistic insights into the molecular interactions contributing to ligand stability within the receptor binding pocket. These findings improve our understanding of the dynamic behavior of protein–ligand interactions at the molecular level. Given that receptor selectivity is a major objective in dopamine receptor drug discovery because of its potential to improve therapeutic efficacy while minimizing adverse effects, an integrated computational workflow combining molecular docking, MD simulations, and MM-PBSA calculations can facilitate the early prioritization of candidate compounds by identifying interaction trends that guide subsequent experimental studies. However, these in silico approaches cannot establish receptor selectivity or pharmacological activity. Rather, they generate mechanistic hypotheses, reveal conserved interaction patterns, and provide a rational framework for selecting compounds for experimental validation. Accordingly, the combined docking, MD, and MM-PBSA analyses presented herein should be regarded as hypothesis-generating tools that support and inform future receptor-binding and functional studies rather than as conclusive evidence of biological activity (Table 4).

**Table 4:** Components of the binding free energy (kcal/mol) for protein–ligand complexes determined by MM-PBSA

Parameters	D2R						
	AM-0	M4B	M5B	M6B	AM-G	ETI	BMM
ΔE_vdw_	− 31.62	− 37.22	− 32.78	− 33.66	− 37.42	− 31.64	− 32.31
ΔE_ele_	− 3	− 20.31	− 17.16	− 21.09	− 0.41	1.24	− 18.47
ΔE_pol_	11.91	20.25	16.97	19.37	9.09	5.17	21.01
ΔE_np_	− 4.33	− 5.68	− 5.13	− 5.26	− 5.11	− 4.74	− 5.13
ΔH	− 27.04 ± 5	− 42.96 ± 3.5	− 38.1 ± 2.8	− 40.64 ± 2.7	− 33.85 ± 3.9	− 29.97	− 34.9 ± 2.9
− TΔS	17.91	11.31	9.0	6.6	11.21	18.33	4.1
ΔG_bind_	− 9.13 ± 3.6	− 31.65 ± 3.5	− 29.08 ± 2.8	− 34.04 ± 2.7	− 22.64 ± 3.9	− 11.64 ± 3.5	30.8 ± 2.9
	D3R						
ΔE_vdw_	− 39.8	− 33.35	− 33.09	− 34.81	− 36.98	− 43.27	− 32.04
ΔE_ele_	− 14.26	− 44.59	− 34.29	− 46.74	− 29.95	− 42.6	− 28.38
ΔE_pol_	20.14	43.04	35.79	49.38	36.53	46.65	34.62
ΔE_np_	− 5.66	− 5.16	− 5.5	− 5.41	− 5.52	− 6.17	− 4.95
ΔH	− 39.58 ± 3.4	− 40.06 ± 3.0	− 37.09 ± 2.9	− 37.58 ± 5.8	− 35.92 ± 3.2	− 45.39 ± 2.8	− 30.74 ± 2.5
− TΔS	12.86	10.55	10.69	5.87	16.23	14.91	16.5
ΔG_bind_	− 26.7 ± 3.8	− 29.51 ± 3.0	− 26.4 ± 2.9	− 31.71 ± 2.0	− 19.69 ± 3.2	− 30.48 ± 2.8	− 14.55 ± 2.5
	D4R						
ΔE_vdw_	− 35.05	− 36.97	− 43.89	− 40.31	− 34.8	− 44.99	− 31.23
ΔE_ele_	− 14.38	− 39.84	− 24.05	− 29.55	− 25.6	− 34.99	− 44.7
ΔE_pol_	21.38	40.43	33.29	37.53	29.08	39.94	47.47
ΔE_np_	− 4.83	− 5.66	− 6.3	− 5.9	− 5.23	− 6.5	− 5.14
ΔH	− 32.88 ± 3.2	− 42.04 ± 2.4	− 40.95 ± 2.6	− 38.23 ± 2.7	− 36.55 ± 3.8	− 46.54 ± 2.6	− 33.6 ± 2.4
− TΔS	28.21	10.58	10.49	22.01	15.09	5.1	9.3
ΔG_bind_	− 4.69 ± 3.2	− 31.46 ± 2.4	− 30.46 ± 2.7	− 16.22 ± 2.7	− 7.27 ± 4.2	− 30.45 ± 2.6	− 24.21 ± 2.4

## Conclusion

This study demonstrates the utility of an integrated computational workflow for identifying and prioritizing amathamide G derivatives with favorable interaction profiles across the D2-like dopamine receptor family, thereby providing a rational framework for future experimental evaluation. Using molecular docking, molecular dynamics (MD) simulations, and MM-PBSA free-energy calculations, we comparatively evaluated the interactions of amathamide G (AM-G), its monobrominated derivatives (M4B, M5B, and M6B), and the debrominated derivative (AM-0) with the dopamine receptors D2R, D3R, and D4R. These compounds, which share a benzene–linker–pentacycle scaffold with the reference ligands ETI and BMM, were systematically analyzed to characterize their interaction patterns and dynamic behavior within the receptor binding pockets.

The results provide a comprehensive overview of the structural and dynamic properties of protein–ligand complexes, highlighting key interactions that contribute to binding stability. In particular, molecular dynamics and MM-PBSA analyses suggested that van der Waals and hydrophobic interactions play a major role in ligand stabilization, whereas hydrogen bonding alone does not fully account for binding affinity.

Drug-likeness and pharmacokinetic studies suggested that debromination of AM-G may influence physicochemical and ADME-related properties, potentially improving their suitability for central nervous system (CNS)-related applications. Overall, M4B, M5B, M6B and AM-0 exhibited several predicted improvements relative to the parent compound AM-G, supporting their prioritization for future experimental studies.

Comparative analyses consistently identified receptor-dependent interaction trends across the D2-like receptor family. Several derivatives exhibited comparatively favorable interaction profiles depending on the receptor subtype evaluated, suggesting that structural modifications such as bromination patterns may influence receptor–ligand interactions within the computational framework employed. However, these observations should be interpreted as relative computational trends useful for compound prioritization rather than evidence of pharmacological selectivity, agonist/antagonist behavior, or absolute binding affinity. Experimental receptor-binding and functional studies will be required to validate these predictions.

In general, this study highlights the value of integrated in silico approaches for exploring ligand-receptor interactions and guiding rational early-stage drug discovery effort. The findings presented herein provide a rationale for prioritizing selected AM-G derivatives for subsequent experimental characterization, including receptor-binding, functional, pharmacology, and toxicological studies, aimed at establishing their biological activity and therapeutic relevance. Beyond the identification of promising lead compounds, this work illustrates how integrated computational approaches can bridge medicinal chemistry and structural pharmacology to generate experimentally testable hypotheses for GPCR-targeted drug discovery.

## Supplementary Information

Below is the link to the electronic supplementary material.

Supplementary Material 1

## Data Availability

No datasets were generated or analysed during the current study.
