# Supplementary material for: Design, synthesis, drug-likeness, and molecular dynamics evaluations of monobrominated amathamide G analogs as potential ligands for D2-like dopamine receptors
Source: In Silico Pharmacol. 2026 Sep 6;14(3):233. doi: 10.1007/s40203-026-00741-1 (PMC13547561; doi:10.1007/s40203-026-00741-1)
Supplement: Supplementary file 1 [file 40203_2026_741_MOESM1_ESM.docx]

**Supplementary Information**

**Design, synthesis, drug likeness and molecular dynamics evaluations of monobrominated Amathamide G analogs as potential inhibitors of D2-like dopamine receptors**

Angelica Navarrete-Gutiérrez^1^, Octavio Frias-Aviña^1^, Julio Montes-Ávila^2^, Raúl Argüello-García^3^, Edar O. Pech-Santiago^3,^*, Gerardo Aguirre-Hernández^1,^*

^a^ Centro de Graduados e Investigación en Química, Tecnológico Nacional de México/IT de Tijuana, Tijuana, B.C., México

^b^ Facultad de Ciencias Químico Biológicas, Universidad Autónoma de Sinaloa, Culiacán, Sin., México

^c^ Departamento de Genética y Biología Molecular, Centro de Investigación y de Estudios Avanzados del Instituto Politécnico Nacional, Mexico City, Mexico.

**Table of contents**

| **Fig. S1**. ^1^H NMR spectrum of (*E*)-1,2-dimethoxy-3-(2-nitrovinyl)benzene (**2a**) | 3 |
| --- | --- |
| **Fig. S2**. ^13^C NMR spectrum of (*E*)-1,2-dimethoxy-3-(2-nitrovinyl)benzene (**2a**) | 3 |
| **Fig. S3**. ^1^H NMR spectrum of (*E*)-1-bromo-3,4-dimethoxy-2-(2-nitrovinyl) benzene (**2b**) | 4 |
| **Fig. S4**. ^13^C NMR spectrum of (*E*)-1-bromo-3,4-dimethoxy-2-(2-nitrovinyl) benzene (**2b**) | 4 |
| **Fig. S5**. ^1^H NMR spectrum of (*E*)-5-bromo-1,2-dimethoxy-3-(2-nitrovinyl) benzene (**2c**) | 5 |
| **Fig. S6**. ^13^C NMR spectrum of (*E*)-5-bromo-1,2-dimethoxy-3-(2-nitrovinyl) benzene (**2c**) | 5 |
| **Fig. S7**. ^1^H NMR spectrum of (*E*)-1-bromo-2,3-dimethoxy-4-(2-nitrovinyl) benzene (**2d**) | 6 |
| **Fig. S8**. ^13^C NMR spectrum of (*E*)-1-bromo-2,3-dimethoxy-4-(2-nitrovinyl) benzene (**2d**) | 6 |
| **Fig. S9**. ^1^H NMR spectrum of (1-(2,3-dimethoxyphenyl)-2-nitroethyl)(phenyl) sulfane (**3a**) | 7 |
| **Fig. S10**. ^13^C NMR spectrum of (1-(2,3-dimethoxyphenyl)-2-nitroethyl)(phenyl) sulfane (**3a**) | 7 |
| **Fig. S11**. ^1^H NMR spectrum of (1-(6-bromo-2,3-dimethoxyphenyl)-2-nitroethyl) (phenyl)sulfane (**3b**) | 8 |
| **Fig. S12**. ^13^C NMR spectrum of (1-(6-bromo-2,3-dimethoxyphenyl)-2-nitroethyl)(phenyl)sulfane (**3b**) | 8 |
| **Fig. S13**. ^1^H NMR spectrum of (1-(5-bromo-2,3-dimethoxyphenyl)-2-nitroethyl)(phenyl)sulfane (**3c**) | 9 |
| **Fig. S14**. ^13^C NMR spectrum of (1-(5-bromo-2,3-dimethoxyphenyl)-2-nitroethyl)(phenyl)sulfane (**3c**) | 9 |
| **Fig. S15**. ^1^H NMR spectrum of (1-(4-bromo-2,3-dimethoxyphenyl)-2-nitroethyl)(phenyl)sulfane (**3d**) | 10 |
| **Fig. S16**. ^13^C NMR spectrum of (1-(4-bromo-2,3-dimethoxyphenyl)-2-nitroethyl)(phenyl)sulfane (**3d**) | 10 |
| **Fig. S17**. ^1^H NMR spectrum of (2*S*)-*N*-(2-(2,3-dimethoxyphenyl)-2-(phenylthio)ethyl)-1-methylpyrrolidine-2-carboxamide (**5a**) | 11 |
| **Fig. S18**. ^13^C NMR spectrum of (2*S*)-*N*-(2-(2,3-dimethoxyphenyl)-2-(phenylthio)ethyl)-1-methylpyrrolidine-2-carboxamide (**5a**) | 11 |
| **Fig. S19**. ^1^H NMR spectrum of (2*S*)-*N*-(2-(6-bromo-2,3-dimethoxyphenyl)-2-(phenylthio)ethyl)-1-methylpyrrolidine-2-carboxamide (**5b**) | 12 |
| **Fig. S20**. ^13^C NMR spectrum of (2*S*)-*N*-(2-(6-bromo-2,3-dimethoxyphenyl)-2-(phenylthio)ethyl)-1-methylpyrrolidine-2-carboxamide (**5b**) | 12 |
| **Fig. S21**. ^1^H NMR spectrum of (2*S*)-*N*-(2-(5-bromo-2,3-dimethoxyphenyl)-2-(phenylthio)ethyl)-1-methylpyrrolidine-2-carboxamide (**5c**) | 13 |
| **Fig. S22**. ^13^C NMR spectrum of (2*S*)-*N*-(2-(5-bromo-2,3-dimethoxyphenyl)-2-(phenylthio)ethyl)-1-methylpyrrolidine-2-carboxamide (**5c**) | 13 |
| **Fig. S23**. ^1^H NMR spectrum of (2*S*)-*N*-(2-(4-bromo-2,3-dimethoxyphenyl)-2-(phenylthio)ethyl)-1-methylpyrrolidine-2-carboxamide (**5d**) | 14 |
| **Fig. S24**. ^13^C NMR spectrum of (2*S*)-*N*-(2-(4-bromo-2,3-dimethoxyphenyl)-2-(phenylthio)ethyl)-1-methylpyrrolidine-2-carboxamide (**5d**) | 14 |
| **Fig. S25**. ^1^H NMR spectrum of (*S*,*E*)-N-(2,3-dimethoxystyryl)-1-methylpyrrolidine-2-carboxamide (**6a**) | 15 |
| **Fig. S26**. ^13^C NMR spectrum of (*S*,*E*)-*N*-(2,3-dimethoxystyryl)-1-methylpyrrolidine-2-carboxamide (**6a**) | 15 |
| **Fig. S27**. ^1^H NMR spectrum of (*S*,*E*)-*N*-(6-bromo-2,3-dimethoxystyryl)-1-methylpyrrolidine-2-carboxamide (**6b**) | 16 |
| **Fig. S28**. ^13^C NMR spectrum of (*S*,*E*)-*N*-(6-bromo-2,3-dimethoxystyryl)-1-methylpyrrolidine-2-carboxamide (**6b**) | 16 |
| **Fig. S29**. ^1^H NMR spectrum of (*S*,*E*)-*N*-(5-bromo-2,3-dimethoxystyryl)-1-methylpyrrolidine-2-carboxamide (**6c**) | 17 |
| **Fig. S30**. ^13^C NMR spectrum of (*S*,*E*)-*N*-(5-bromo-2,3-dimethoxystyryl)-1-methylpyrrolidine-2-carboxamide (**6c**) | 17 |
| **Fig. S31**. ^1^H NMR spectrum of (S,E)-*N*-(4-bromo-2,3-dimethoxystyryl)-1-methylpyrrolidine-2-carboxamide (**6d**) | 18 |
| **Fig. S32**. ^13^C NMR spectrum of (S,E)-*N*-(4-bromo-2,3-dimethoxystyryl)-1-methylpyrrolidine-2-carboxamide (**6d**) | 18 |
| **Fig. S33**. EI-MS spectrum of (*E*)-1,2-dimethoxy-3-(2-nitrovinyl)benzene (**2a**) | 19 |
| **Fig. S34**. EI-MS spectrum of (*E*)-1-bromo-3,4-dimethoxy-2-(2-nitrovinyl) benzene (**2b**) | 19 |
| **Fig. S35**. EI-MS spectrum of (*E*)-5-bromo-1,2-dimethoxy-3-(2-nitrovinyl) benzene (**2c**) | 20 |
| **Fig. S36**. EI-MS spectrum of (*E*)-1-bromo-2,3-dimethoxy-4-(2-nitrovinyl) benzene (**2d**) | 20 |
| **Fig. S37**. EI-MS spectrum of (1-(2,3-dimethoxyphenyl)-2-nitroethyl)(phenyl) sulfane (**3a**) | 21 |
| **Fig. S38**. EI-MS spectrum of (1-(6-bromo-2,3-dimethoxyphenyl)-2-nitroethyl) (phenyl)sulfane (**3b**) | 21 |
| **Fig. S39**. EI-MS spectrum of (1-(4-bromo-2,3-dimethoxyphenyl)-2-nitroethyl)(phenyl)sulfane (**3d**) | 22 |
| **Fig. S40**. EI-MS spectrum of (2*S*)-*N*-(2-(2,3-dimethoxyphenyl)-2-(phenylthio)ethyl)-1-methylpyrrolidine-2-carboxamide (**5a**) | 22 |
| **Fig. S41**. EI-MS spectrum of (2*S*)-*N*-(2-(6-bromo-2,3-dimethoxyphenyl)-2-(phenylthio)ethyl)-1-methylpyrrolidine-2-carboxamide (**5b**) | 23 |
| **Fig. S42**. EI-MS spectrum of (2*S*)-*N*-(2-(5-bromo-2,3-dimethoxyphenyl)-2-(phenylthio)ethyl)-1-methylpyrrolidine-2-carboxamide (**5c**) | 23 |
| **Fig. S43**. EI-MS spectrum of (2*S*)-*N*-(2-(4-bromo-2,3-dimethoxyphenyl)-2-(phenylthio)ethyl)-1-methylpyrrolidine-2-carboxamide (**5d**) | 24 |
| **Fig. S44**. EI-MS spectrum of (*S*,*E*)-N-(2,3-dimethoxystyryl)-1-methylpyrrolidine-2-carboxamide (**6a**) | 24 |
| **Fig. S45**. EI-MS spectrum of (*S*,*E*)-*N*-(6-bromo-2,3-dimethoxystyryl)-1-methylpyrrolidine-2-carboxamide (**6b**) | 25 |
| **Fig. S46**. EI-MS spectrum of (*S*,*E*)-*N*-(5-bromo-2,3-dimethoxystyryl)-1-methylpyrrolidine-2-carboxamide (**6c**) | 25 |
| **Fig. S47**. EI-MS spectrum of (S,E)-*N*-(4-bromo-2,3-dimethoxystyryl)-1-methylpyrrolidine-2-carboxamide (**6d**)  **Fig. S48**. RMSD values and superposition of of cristal (magenta) and docked (green) structure of D2-like. (A) D2R with risperidone, (B) D3R with eticlopride, and (C) D4R with nemonapride. | 26 |
| **Table S1.** Physicochemical properties of AM-G, its monobrominated (M6B. M5B. M4B) and debrominated (AM-0) derivatives, the standard compound Eticlopride and the reference compound BMM. | 27 |

**Fig. S1**. ^1^H NMR spectrum of (*E*)-1,2-dimethoxy-3-(2-nitrovinyl)benzene (**2a**)

**Fig. S2**. ^13^C NMR spectrum of (*E*)-1,2-dimethoxy-3-(2-nitrovinyl)benzene (**2a**)

**Fig. S3**. ^1^H NMR spectrum of (*E*)-1-bromo-3,4-dimethoxy-2-(2-nitrovinyl) benzene (**2b**)

**Fig. S4**. ^13^C NMR spectrum of (*E*)-1-bromo-3,4-dimethoxy-2-(2-nitrovinyl) benzene (**2b**)

**Fig. S5**. ^1^H NMR spectrum of (*E*)-5-bromo-1,2-dimethoxy-3-(2-nitrovinyl) benzene (**2c**)

**Fig. S6**. ^13^C NMR spectrum of (*E*)-5-bromo-1,2-dimethoxy-3-(2-nitrovinyl) benzene (**2c**)

**Fig. S7**. ^1^H NMR spectrum of (*E*)-1-bromo-2,3-dimethoxy-4-(2-nitrovinyl) benzene (**2d**)

**Fig. S8**. ^13^C NMR spectrum of (*E*)-1-bromo-2,3-dimethoxy-4-(2-nitrovinyl) benzene (**2d**)

**Fig. S9**. ^1^H NMR spectrum of (1-(2,3-dimethoxyphenyl)-2-nitroethyl)(phenyl) sulfane (**3a**)

**Fig. S10**. ^13^C NMR spectrum of (1-(2,3-dimethoxyphenyl)-2-nitroethyl)(phenyl) sulfane (**3a**)

**Fig. S11**. ^1^H NMR spectrum of (1-(6-bromo-2,3-dimethoxyphenyl)-2-nitroethyl) (phenyl)sulfane (**3b**)

**Fig. S12**. ^13^C NMR spectrum of (1-(6-bromo-2,3-dimethoxyphenyl)-2-nitroethyl)(phenyl)sulfane (**3b**)

**Fig. S13**. ^1^H NMR spectrum of (1-(5-bromo-2,3-dimethoxyphenyl)-2-nitroethyl)(phenyl)sulfane (**3c**)

**Fig. S14**. ^13^C NMR spectrum of (1-(5-bromo-2,3-dimethoxyphenyl)-2-nitroethyl)(phenyl)sulfane (**3c**)

**Fig. S15**. ^1^H NMR spectrum of (1-(4-bromo-2,3-dimethoxyphenyl)-2-nitroethyl)(phenyl)sulfane (**3d**)

**Fig. S16**. ^13^C NMR spectrum of (1-(4-bromo-2,3-dimethoxyphenyl)-2-nitroethyl)(phenyl)sulfane (**3d**)

**Fig. S17**. ^1^H NMR spectrum of (2*S*)-*N*-(2-(2,3-dimethoxyphenyl)-2-(phenylthio)ethyl)-1-methylpyrrolidine-2-carboxamide (**5a**)

**Fig. S18**. ^13^C NMR spectrum of (2*S*)-*N*-(2-(2,3-dimethoxyphenyl)-2-(phenylthio)ethyl)-1-methylpyrrolidine-2-carboxamide (**5a**)

**Fig. S19**. ^1^H NMR spectrum of (2*S*)-*N*-(2-(6-bromo-2,3-dimethoxyphenyl)-2-(phenylthio)ethyl)-1-methylpyrrolidine-2-carboxamide (**5b**)

**Fig. S20**. ^13^C NMR spectrum of (2*S*)-*N*-(2-(6-bromo-2,3-dimethoxyphenyl)-2-(phenylthio)ethyl)-1-methylpyrrolidine-2-carboxamide (**5b**)

**Fig. S21**. ^1^H NMR spectrum of (2*S*)-*N*-(2-(5-bromo-2,3-dimethoxyphenyl)-2-(phenylthio)ethyl)-1-methylpyrrolidine-2-carboxamide (**5c**)

**Fig. S22**. ^13^C NMR spectrum of (2*S*)-*N*-(2-(5-bromo-2,3-dimethoxyphenyl)-2-(phenylthio)ethyl)-1-methylpyrrolidine-2-carboxamide (**5c**)

**Fig. S23**. ^1^H NMR spectrum of (2*S*)-*N*-(2-(4-bromo-2,3-dimethoxyphenyl)-2-(phenylthio)ethyl)-1-methylpyrrolidine-2-carboxamide (**5d**)

**Fig. S24**. ^13^C NMR spectrum of (2*S*)-*N*-(2-(4-bromo-2,3-dimethoxyphenyl)-2-(phenylthio)ethyl)-1-methylpyrrolidine-2-carboxamide (**5d**)

**Fig. S25**. ^1^H NMR spectrum of (*S*,*E*)-N-(2,3-dimethoxystyryl)-1-methylpyrrolidine-2-carboxamide (**6a**)

**Fig. S26**. ^13^C NMR spectrum of (*S*,*E*)-*N*-(2,3-dimethoxystyryl)-1-methylpyrrolidine-2-carboxamide (**6a**)

**Fig. S27**. ^1^H NMR spectrum of (*S*,*E*)-*N*-(6-bromo-2,3-dimethoxystyryl)-1-methylpyrrolidine-2-carboxamide (**6b**)

**Fig. S28**. ^13^C NMR spectrum of (*S*,*E*)-*N*-(6-bromo-2,3-dimethoxystyryl)-1-methylpyrrolidine-2-carboxamide (**6b**)

**Fig. S29**. ^1^H NMR spectrum of (*S*,*E*)-*N*-(5-bromo-2,3-dimethoxystyryl)-1-methylpyrrolidine-2-carboxamide (**6c**)

**Fig. S30**. ^13^C NMR spectrum of (*S*,*E*)-*N*-(5-bromo-2,3-dimethoxystyryl)-1-methylpyrrolidine-2-carboxamide (**6c**)

**Fig. S31**. ^1^H NMR spectrum of (S,E)-*N*-(4-bromo-2,3-dimethoxystyryl)-1-methylpyrrolidine-2-carboxamide (**6d**)

**Fig. S32**. ^13^C NMR spectrum of (S,E)-*N*-(4-bromo-2,3-dimethoxystyryl)-1-methylpyrrolidine-2-carboxamide (**6d**)

**Fig. S33**. EI-MS spectrum of (*E*)-1,2-dimethoxy-3-(2-nitrovinyl)benzene (**2a**)

**Fig. S34**. EI-MS spectrum of (*E*)-1-bromo-3,4-dimethoxy-2-(2-nitrovinyl) benzene (**2b**)

**Fig. S35**. EI-MS spectrum of (*E*)-5-bromo-1,2-dimethoxy-3-(2-nitrovinyl) benzene (**2c**)

**Fig. S36**. EI-MS spectrum of (*E*)-1-bromo-2,3-dimethoxy-4-(2-nitrovinyl) benzene (**2d**)

**Fig. S37**. EI-MS spectrum of (1-(2,3-dimethoxyphenyl)-2-nitroethyl)(phenyl) sulfane (**3a**)

**Fig. S38**. EI-MS spectrum of (1-(6-bromo-2,3-dimethoxyphenyl)-2-nitroethyl) (phenyl)sulfane (**3b**)

**Fig. S39**. EI-MS spectrum of (1-(4-bromo-2,3-dimethoxyphenyl)-2-nitroethyl)(phenyl)sulfane (**3d**)

**Fig. S40**. EI-MS spectrum of (2*S*)-*N*-(2-(2,3-dimethoxyphenyl)-2-(phenylthio)ethyl)-1-methylpyrrolidine-2-carboxamide (**5a**)

**Fig. S41**. EI-MS spectrum of (2*S*)-*N*-(2-(6-bromo-2,3-dimethoxyphenyl)-2-(phenylthio)ethyl)-1-methylpyrrolidine-2-carboxamide (**5b**)

**Fig. S42**. EI-MS spectrum of (2*S*)-*N*-(2-(5-bromo-2,3-dimethoxyphenyl)-2-(phenylthio)ethyl)-1-methylpyrrolidine-2-carboxamide (**5c**)

**Fig. S43**. EI-MS spectrum of (2*S*)-*N*-(2-(4-bromo-2,3-dimethoxyphenyl)-2-(phenylthio)ethyl)-1-methylpyrrolidine-2-carboxamide (**5d**)

**Fig. S44**. EI-MS spectrum of (*S*,*E*)-N-(2,3-dimethoxystyryl)-1-methylpyrrolidine-2-carboxamide (**6a**)

**Fig. S45**. EI-MS spectrum of (*S*,*E*)-*N*-(6-bromo-2,3-dimethoxystyryl)-1-methylpyrrolidine-2-carboxamide (**6b**)

**Fig. S46**. EI-MS spectrum of (*S*,*E*)-*N*-(5-bromo-2,3-dimethoxystyryl)-1-methylpyrrolidine-2-carboxamide (**6c**)

**Fig. S47**. EI-MS spectrum of (S,E)-*N*-(4-bromo-2,3-dimethoxystyryl)-1-methylpyrrolidine-2-carboxamide (**6d**)

**Fig. S48**. RMSD values and superposition of of cristal (magenta) and docked (green) structure of D2-like. (A) D2R with risperidone, (B) D3R with eticlopride, and (C) D4R with nemonapride.

**Table S1**. Physicochemical properties of AM-G, its monobrominated (M6B. M5B. M4B) and debrominated (AM-0) derivatives, the standard compound Eticlopride and the reference compound BMM.

| Property | AM-G | M6B | M5B | M4B | AM-0 | ETI | BMM |
| --- | --- | --- | --- | --- | --- | --- | --- |
| Molecular Weight | 523.8 | 368.1 | 368.1 | 368.1 | 290.1 | 340.1 | 308.1 |
| Volume | 361.2 | 322.6 | 322.6 | 322.6 | 303.3 | 338.5 | 266.3 |
| Density | 1.45 | 1.14 | 1.14 | 1.14 | 0.95 | 1.01 | 1.15 |
| nHA | 5 | 5 | 5 | 5 | 5 | 5 | 4 |
| nHD | 1 | 1 | 1 | 1 | 1 | 2 | 2 |
| nRot | 6 | 6 | 6 | 6 | 6 | 7 | 6 |
| nRing | 2 | 2 | 2 | 2 | 2 | 2 | 2 |
| maxRing | 6 | 6 | 6 | 6 | 6 | 6 | 6 |
| nHet | 8 | 6 | 6 | 6 | 5 | 6 | 5 |
| fChar | 0 | 0 | 0 | 0 | 0 | 0 | 0 |
| nRig | 13 | 13 | 13 | 13 | 13 | 12 | 11 |
| Flexibility | 0.46 | 0.46 | 0.46 | 0.46 | 0.46 | 0.58 | 0.54 |
| Stereo centers | 1 | 1 | 1 | 1 | 1 | 1 | 0 |
| TPSA | 50.8 | 50.8 | 50.8 | 50.8 | 50.8 | 61.8 | 41.8 |
| logS | -3.66 | -2.58 | -2.50 | -2.33 | -1.58 | -3.14 | -0.45 |
| logP | 3.80 | 2.34 | 2.48 | 2.25 | 1.52 | 3.62 | 1.84 |
| logD | 3.56 | 2.53 | 2.75 | 2.59 | 1.84 | 2.81 | 1.16 |

MW: contains hydrogen atoms. Optimal 100-600; Volume: Van der Waals volumen; Density: MW/volumen; nHA: number of hydrogen bond acceptors. Optimal 0-12; nHD: number of hydrogen bond donors. Optimal 0-7; nRot: number of rotatable bonds. Optimal 0-11; nRing: number of ring. optimal 0-6; MaxRing: number of atoms in the biggest ring. optimal 0-18; nHet: number of heteroatoms. Optimal 0-15; fChar: formal charge. Optimal -4 to 4; nRig: number of rigid bonds. optimal 0-30; Flexibility: nRot/nRig; Stereo Centers: optimal ≤ 2; TPSA: topological polar surface area. Optimal 0-140; logS: log of the aqueous solubility. Optimal -4 to 0.5 log mol/L; logP: logof the octanol/water partition coefficient. Optimal 0-3; logD: logP at physiological pH 7.4. Optimal 1-3.
